# Bmp Signal Gradient Modulates Convergent Cell Movement via *Xarhgef3.2* during Gastrulation of *Xenopus* Embryos

**DOI:** 10.3390/cells11010044

**Published:** 2021-12-24

**Authors:** Jaeho Yoon, Vijay Kumar, Ravi Shankar Goutam, Sung-Chan Kim, Soochul Park, Unjoo Lee, Jaebong Kim

**Affiliations:** 1 Department of Biochemistry, Institute of Cell Differentiation and Aging, College of Medicine, Hallym University, Chuncheon 24252, Korea; jaeho.yoon@nih.gov (J.Y.); vijay10187@gmail.com (V.K.); ravi2005gautam@gmail.com (R.S.G.); biokim@hallym.ac.kr (S.-C.K.); 2 National Cancer Institute, Frederick, MD 21702, USA; 3 Department of Biological Sciences, Sookmyung Women’s University, Seoul 04310, Korea; scpark@sookmyung.ac.kr; 4 Department of Electrical Engineering, Hallym University, Chuncheon 24252, Korea; ejlee@hallym.ac.kr

**Keywords:** Bmp, Wnt-PCP, gastrulation, *Xarhgef3.2*, Disheveled, daam1, convergent extension, *Xenopus*

## Abstract

Gastrulation is a critical step in the establishment of a basic body plan during development. Convergence and extension (CE) cell movements organize germ layers during gastrulation. Noncanonical Wnt signaling has been known as major signaling that regulates CE cell movement by activating Rho and Rac. In addition, Bmp molecules are expressed in the ventral side of a developing embryo, and the ventral mesoderm region undergoes minimal CE cell movement while the dorsal mesoderm undergoes dynamic cell movements. This suggests that Bmp signal gradient may affect CE cell movement. To investigate whether Bmp signaling negatively regulates CE cell movements, we performed microarray-based screening and found that the transcription of *Xenopus* Arhgef3.2 (Rho guanine nucleotide exchange factor) was negatively regulated by Bmp signaling. We also showed that overexpression or knockdown of *Xarhgef3.2* caused gastrulation defects. Interestingly, Xarhgef3.2 controlled gastrulation cell movements through interacting with Disheveled (Dsh2) and Dsh2-associated activator of morphogenesis 1 (Daam1). Our results suggest that Bmp gradient affects gastrulation cell movement (CE) via negative regulation of *Xarhgef3.2* expression.

## 1. Introduction

Cell fate determination and gastrulation-induced movement are two most important events in early embryogenesis. In *Xenopus*, after fertilization, a single-cell embryo undergoes sequential cleavages with the cells at the blastula stage forming three germ layers, referred to as ectoderm, mesoderm, and endoderm [1]. The establishment of these germ layers utilizes several signaling pathways that include Bmp, TGF-β, FGF, and Wnt/β-catenin signaling. Movement of these specialized germ layers to their specific positions marks the onset of gastrulation in a process termed convergence and extension (CE) movements. This phenomenon of gastrulation cell movement is mainly governed by Wnt-planar cell polarity (Wnt-PCP) signaling [2]. Furthermore, during gastrulation, the dorsal mesoderm and neural tissues converge and extend dramatically, pushing the future head tissues away from the tail to form extended anteroposterior body axis [3]. These events occur in an orderly fashion, involving different signaling cascades, but the correlation between these two processes has not been investigated in detail. Cell fate determination for an early vertebrate embryo is predominantly brought about by Bmp gradient signaling in the ventral region and, also in part, by BMP antagonists secreted in the dorsal region of the Spemann organizer [1,4,5]. The Spemann organizer itself maintains dorsoventral patterning by expressing Bmp antagonists such as *chordin* (*chrd*), *noggin* (*nog*), and *follistatin* (*fol*) [6]. For Bmp4, its gradient specifies the ventral posterior zone and inhibits neurogenesis [7,8]; this is mediated by modulating the activities of various R-Smads such as Smad 1/5/8 and regulating target gene expression [9]. There have also been reports in which the Bmp4 signaling gradient modulates CE movement. In zebrafish, for example, the Bmp signaling gradient plays an instructive role in regulating CE movement during gastrula [10]. In addition, overexpression of Bmp inhibits activin-induced CE in *Xenopus* animal caps (ACs) [11], while blocking Bmp can induce CE of the ventral marginal zone [12].

In all, Bmp signaling gradient is a major determinant for asymmetric development during embryogenesis in vertebrates. In *Xenopus* embryos, the Bmp4 gradient is generated by cells located in the embryonic ventral region and decreases towards the dorsal side of an embryo. As for the CE activity, it is strong near the dorsal area and becomes weak near the ventral side of embryos. This arrangement implies that Bmp signaling gradient is somehow negatively involved in the regulation of CE cell movement in *Xenopus*; however, the cause and effects in such a scenario are largely unknown. CE actively occurs in the dorsal mesoderm and the neural ectoderm to narrow the width of these tissues and widen their length along the anteroposterior axis, giving rise to the basic vertebrate body plan. For gastrulation, CE cell movement is precisely regulated by noncanonical Wnt signaling [13,14,15,16], and Wnt-PCP signaling has been described as the major signaling pathway regulating CE. Wnt-PCP signaling utilizes noncanonical Wnt signaling components such as Frizzled (Fz), Dsh2, Dsh2-associated activator of morphogenesis 1 (Daam1), guanine nucleotide exchange factors (RhoGEF), and small GTPases such as RhoA, Rac1, and Cdc42. Rho GTPases have already been shown to be involved in cell adhesion and cell cytokinesis in *Xenopus* [17,18,19,20] and in CE movement during gastrulation [21,22,23,24]. Activation of Rho GTPases is mediated by guanine nucleotide exchange factors (GEFs) [25,26] that catalyze GDP-to-GTP exchange, leading to Rho activation. About 85 RhoGEFs have been identified in vertebrates, and of these, *Xwgef* (*arhgef19*) and *Xnet1* (*arhgef8*) have been studied in *Xenopus* gastrulation cell movement [27,28]. Xwgef and Xnet1 are homologous to mammalian RhoA-specific GEFs. Both overexpression and mutant versions of *Xnet1* critically affect gastrulation cell movement in *Xenopus* [28]. Interestingly, overexpression of *Xwgef* is able to rescue CE suppression triggered by dominant-negative Wnt11, and downregulation of Xwgef leads to inhibition of CE movement in *Xenopus* [27]. An intracellular Wnt signaling element, Dsh2, also plays a major role in the CE process in *Xenopus* [13,14,15,16]. Both Xwgef and Xnet1 control CE movement via interacting with Dsh2 through RhoA activation [27]. Nevertheless, expression of *Xwgef* and *Xnet1* is ubiquitous even in the ventral marginal zone (VMZ) and the animal pole region, although these regions undergo weak CE cell movements. Given many important regulatory processes of *Xenopus* embryogenesis are mediated by Rho GTPases, the regulatory mechanisms of Rho GTPases still remain to be worked out as only few RhoGEFs have been well-characterized in the early development of an organism.

In the current study, we hypothesized that cell fate determination and subsequent gastrulation movement are linked via Bmp gradient, and we aimed to uncover an interconnected Wnt-PCP molecule whose expression is governed by BMP gradient, linking the two events. Our Affymetrix microarray data revealed that several ESTs associated with cell movement were markedly affected by the Bmp4 signaling gradient in the dorsal marginal zone (DMZ) of *Xenopus*. Among these ESTs, Xl.3374 encodes the guanine nucleotide exchange factor for RhoA, Xarhgef3.2.L (Xarhgef3.2), and is similar to human Arhgef3 [29,30]. Compared to *Xwgef* and *Xnet1*, endogenous expression of *Xarhgef3.2* was progressively upregulated by Bmp4 inhibition in ACs of the gastrula stage. Overexpression and knockdown of *Xarhgef3.2* severely impaired gastrulation movement in the *Xenopus* embryos. We also show that Xarhgef3.2 activates RhoA but not Rac1 or Cdc42 via a physical interaction with Dsh2 and Daam1. Taken together, our data point to Xarhgef3.2 as a key regulator of CE cell movement, its expression being negatively modulated by the Bmp4 signaling gradient. Thus, we propose that BMP4 signaling negatively regulates gastrulation cell movement by modulating transcription of *Xarhgef3.2*, a noncanonical Wnt component gene, during the early gastrula of *Xenopus* embryos.

## 2. Materials and Methods

### 2.1. Ethics Statement

The animal studies were conducted in accordance with the approved protocols and oversight from the Institutional Animal Care and Use Committee (IACUC) of Hallym University (Hallym 2019-79, 2019-80). All our research team members attended educational and training courses for the appropriate care and use of experimental animals. Adult *X. laevis* were maintained in suitable containers under a 12 h light/dark (LD 12:12 h) cycle at 18 °C, tended by authorized personnel, according to the guidelines of the Institute of Laboratory Animal Resources of Hallym University.

### 2.2. Embryos Injection and Explant Culture

*Xenopus* laevis were obtained from the Korean *Xenopus* Resource Center for Research (Seoul, Korea). The embryos were injected following in vitro fertilization of eggs [31], the frogs having been induced by injection of 500 units of human chorionic gonadotropin (Sigma, St. Louis, MI, USA). RNAs were injected into the embryo animal pole at the one- or two-cell stage and then cultured in 30% Marc’s Modified Ringer’s (MMR) solution. RNAs of β-galactosidase (1 ng/embryo) or eGFP (1 ng/embryo) were injected as control to check for gastrulation defects at stage 11 [32]. Developmental stages were designated according to the Nieuwkoop and Faber Normal Table of *Xenopus laevis* (Daudin) [33]. Animal caps (ACs) were then dissected from the injected and noninjected embryos at stage 8.0–8.5 and incubated in 1× L-15 growth medium (Gibco/Thermo Fisher, Waltham, MA, USA) until stages 11 and 24 in preparation for RT-PCR.

### 2.3. Sample Preparation and Microarray Analysis

A dominant negative BMP type I receptor (DNBR) (lacking the serine/threonine kinase domain) [34,35] RNA (500 pg/embryo) was injected into the embryo animal pole at the one- or two-cell stage and then cultured in 30% MMR solution. Animal caps (ACs) were dissected from the injected and noninjected embryos at stage 8–9 and cultured until stage 11 (control and DNBR ACs) [36]. ACs from the noninjected embryos were also cultured to stage 11 with activin (25 ng/mL) or basic Fgf (bFgf, 100 ng/mL) (activin vs. Fgf ACs) in 67% Leibovitz’s L-15 medium (Gibco, Waltham, MA, USA) supplemented with 0.3 mg/mL L-glutamine, 7 mM Tris-HCl, pH 7.5, and 50 μg/mL gentamicin. For each microarray experiment, about 500 ACs were harvested and stored in RNA*later* (Qiagen, Hilden, Germany), an RNA stabilization reagent, at 4 °C until RNA extraction. Total RNA was extracted from four groups of the ACs (control, DNBR, activin, and Fgf) with an RNeasy Mini kit (Qiagen, Hilden, Germany) following the manufacturer’s instructions. Microarray experiments were performed by Seoulin Bioscience (Seoul, Korea) with an Affymetrix *Xenopus* Genome Gene Chip (Affymetrix) and described on their website (www.seoulin.co.kr, accessed on 25 June 2011).

### 2.4. Microarray Data Normalization, Analysis, and Phylogenetic Tree

Signals from the four groups of samples were normalized and analyzed by Seoulin Bioscience (Seoul, Korea). We presented the gene lists of transcripts showing a higher than log_2_-fold change when compared with the log_2_ value of the control signal (see the Supplementary Tables). The selected EST gene was used to perform the experiments [36,37]. The phylogenetic tree of arhgef3 and orthologs was built using Clustal Omega (https://www.ebi.ac.uk/Tools/msa/clustalo/ (accessed on 16 December 2021) and iTOL (https://itol.embl.de/, accessed on 16 December 2021) [38]. The protein sequences in the FASTA format were downloaded from Uniprot (https://www.uniprot.org/, accessed on 15 December 2021). The Supplementary Tables provides details of protein sequences and accession numbers.

### 2.5. RNA Isolation and Reverse Transcription Polymerase Chain Reaction (RT-PCR)

Total RNA was extracted from either the whole embryos or the cultured ACs with the TRIzol reagent (Life Technologies, Carlsbad, CA, USA) following the manufacturer’s instructions. RT-PCR was performed according to the following parameters: denaturation at 94 °C for 5 min, 19–30 cycles of melting at 94 °C for 1 min, annealing at a given temperature for 1 min, and extension at 72 °C for 1 min. Amplification and primer conditions were as described at the *Xenopus* Molecular Marker Resource (XMMR; University of Texas, Austin, TX, USA) unless mentioned in Table 1. ODC or EF1α was used as a control to normalize the amount of cDNA used. The details on the primers are given in Table 1.

### 2.6. In Vitro Transcription

The *Xarhgef3.2* mRNA used for microinjection was produced by in vitro transcription. PCR-amplified *Xarhgef3.2.L* (*Xarhgef3.2.*) cDNA was inserted using the EcoRI and XhoI sites of the pCS2+ vector. The cDNAs were linearized using Asp718 and used for in vitro synthesis of capped mRNA using in an vitro transcription kit (Ambion, Austin, TX, USA) in accordance with the manufacturer’s instructions. The synthetic RNA was quantified by means of ethidium bromide staining, compared with standard RNA.

### 2.7. Embryos and Whole-Mount In Situ Hybridization

Wild-type and albino *Xenopus laevis* embryos were obtained by hormone-induced egg laying and in vitro fertilization using standard methods. Whole-mount in situ hybridization was performed as described previously [42], with modifications [43]. For double-staining analysis, digoxigenin-UTP- and fluorescein-UTP-labeled RNA probes were used. After the first staining with NBT/BCIP, the enzyme reaction was stopped by heating the embryos for 20 min in 0.1× MBS supplemented with 10 M EDTA. Staining for the second transcript was as for the first one, but using Fast Red (Boehringer Mannheim, Mannheim, Germany) as a dye. The probes were prepared using digoxigenin or fluorescein RNA-labeling mixes (Boehringer Mannheim) and subsequently purified using an in vitro transcription kit (Ambion, Austin, TX, USA). The probes used were *Xarhgef3.2*, cut with EcoRI and transcribed with the T7 RNA polymerase.

### 2.8. Morpholino Oligos

Morpholino oligos (Gene Tools, Philomath, OR, USA) as antisense oligodeoxynucleotides were used for loss-of-function studies. The base composition of the antisense oligodeoxynucleotide was the 25-mer morpholino, 5′CTG GCA GGT TCA CTG GTC ACA ATT A 3′ (*Xarhgef3.2* MO), which targeted the 5′ UTR region of the *Xarhgef3.2.L* mRNA. The specificity of MO was examined by means of Western blot detection of the Flagged protein after injection of the *5′UTR-Xarhgef3.2-3Flag* construct. The MO was against the 5′UTR region of *5′UTR-Xarhgef3.2* (including the endogenous target). The addback 5′-3Flag version was tagged in its 5′ region and its message would not bind the MO and thus would be expressed. The MO was confirmed to block target *5′UTR-Xarhgef3.2-3Flag* without affecting amounts of endogenous *Xarhgef3.2* RNA.

## 3. Results

### 3.1. Bmp Signal Gradient Modulates Xarhgef3.2 Transcription

**Figure 1:**
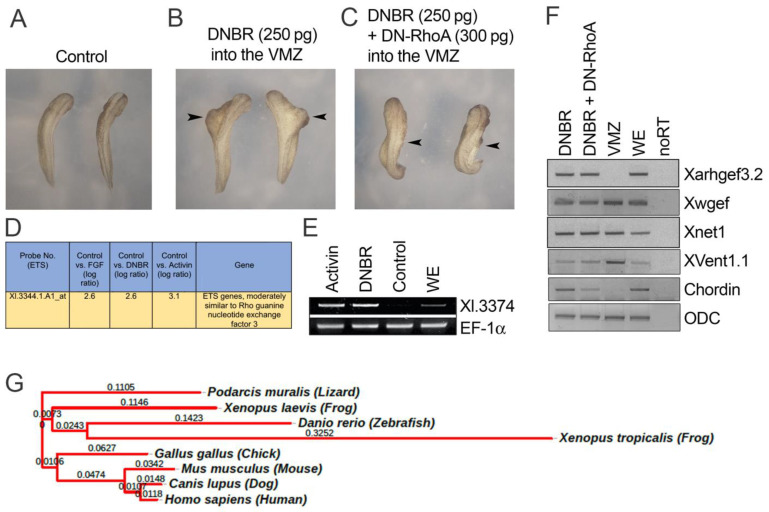
Bmp inhibition induced a secondary axis and requires RhoA activation of gastrulation cell movement in the VMZ. Identification of *Xarhgef3.2*. (**A**–**C**) The embryos were co-injected with 500 pg of DNBR mRNA with or without 300 pg of DN-RhoA at the four-cell stage into the VMZ. The noninjected control and injected embryos were analyzed at the 28–30 stage. (**D**) Microarray analysis data showed that Xl.3374.1.1A_at is induced by DNBR (500 pg/embryo), activin (25 ng/mL), and bFgf (100 ng/mL) at stage 11. (**E**) The microarray data were confirmed by RT-PCR at stage 11; EF-1a was used as a loading control. (**F**) The VMZ explants of the embryos ((**A**–**C**) conditions) were dissected at stage 10. RT-PCR was performed to examine the expression pattern of the indicated genes at stage 12. (**G**) Phylogenetic tree created by amino acid sequence comparison of Xarhgef3.2 of the various species shown in the tree.

In *Xenopus*, Bmp inhibition in the VMZ produces a partial secondary axis in the whole embryo [44]. To examine the involvement of CE movement in partial axis formation by BMP inhibition, the role of Bmp, an essential component of CE movement, was examined using the dominant negative mutant forms of the Bmp receptor (DNBR) and RhoA (DN-RhoA). *Dnbr* (500 pg/embryo) was injected in the VMZ of the four-cell stage embryo, and the injection in the VMZ produced a partial secondary axis (Figure 1B). To examine the involvement of CE movement using a Rho downstream of the Wnt-PCP component [45], *dnbr* (500 pg/embryo) and *DN-RhoA* (300 pg/embryo) were co-injected in the VMZ region of the four-cell stage embryos. As expected, the partial secondary axis formed by inhibition of Bmp disappeared by co-injection of *DN-RhoA* (Figure 1C). Another experiment using the X-gal tracer suggested that formation of a partial secondary axis may require fate change of VMZ cells as well as activation of Wnt-PCP signaling in those cells since BMP-inhibited cells of the VMZ remained part of an embryo without producing a secondary axis in the embryo by *DN-RhoA* co-injection (Supplementary Figure S1A). We confirmed that BMP inhibition in the VMZ led to the conversion of the VMZ to the DMZ in character. The VMZ explants injected with DNBR resulted in a similar elongation phenotype for that of the DMZ explants (Supplementary Figure S1B). The expression levels of the organizer (dorsal mesoderm)-specific gene, *chordin*, were increased; at the same time, the levels of the ventral specific gene, *ventx1.1*, were decreased, and there was no change in the expression of the pan-mesoderm marker, *xbra* (Supplementary Figure S1B).

Activin and Fgf are well-known signal molecules that can promote elongation, mimicking the CE movement in the *Xenopus* AC system [44,46]. In contrast, overexpression of Bmp blocks activin-mediated elongation of ACs [11]. We assumed that the elongation behavior of ACs may be related to expression changes in the Wnt-PCP component genes. To identify factors of the Wnt-PCP pathway involved in BMP inhibition and activin/Fgf-mediated AC elongation (CE movement), three different conditions were assayed in the AC system, followed by profiling of the RNA transcripts isolated from the ACs (stage 11). The conditions were for samples treated either with activin (25 ng/mL), bFgf (100 ng/mL), or those that were dissected from the DNBR-injected embryos (2 ng/embryo). Microarray analysis was performed using RNA from DNBR-injected/activin/bFgf-treated ACs. Gene expression profiling was with a *Xenopus* Affymetrix Gene Chip containing 14,400 gene transcripts for identification of candidate genes. The candidate genes had expression increases under all three AC groups (Figure 1D plus Supplementary Tables). Upregulation of the candidate EST ‘Xl.3374.1.A1_at’ was then confirmed via RT-PCR in RNA obtained from the ACs (stage 11) both for DNBR- (500 pg/embryo) and activin (50 ng/mL)-treated samples (Figure 1E). The cDNA of Xl.3374.1.A1_at encoded Arhgef3.2, which is a Dbl family Rho GEF containing a Dbl homology (DH) domain (mediating GEF activity) and a pleckstrin homology (PH) domain (used for binding). Protein sequence analysis showed a high degree of sequence homology between the *Xenopus* and human versions (at 79.8% identity) (Supplementary Figure S2), and we referred to the gene as *Xarhgef3* (specifically, *Xarhgef3.2.L*). To examine whether *Xarhgef3.2* expression and/or fate change of the VMZ cells were affected in the DNBR and DN-RhoA co-injection group, RT-PCR analysis was performed using the VMZ explants. DN-RhoA co-injection had no additional effects on RhoGEF expression, including for *Xarhgef3.2* and known genes *Xwgef* and *Xnet1.* In addition, obvious cell fate change was not observed, as shown in Figure 1F. Notice that DN-RhoA co-injection still maintained the DMZ character with respect to *chordin* expression (compare the second lane with the third lane of the VMZ in Figure 1F and Supplementary Figure S1C). There was also robust *Xarhgef3.2* expression, indicating that fate change may not be the main reason for the secondary partial axis loss in DN-RhoA- and DNBR-co-injected embryos. Finally, the Xarhgef3.2 protein was conserved, and we presented the phylogenetic tree (Figure 1G) created by amino acid sequence comparison of Xarhgef3.2 shown in Supplementary Figure S2. Altogether, *Xenopus arhgef3.2* upregulation of ACs in both Bmp inhibition and activin-treated conditions suggested that Bmp signal gradient and CE movement may be interlinked through the transcription regulation of *Xarhgef3.2*.

### 3.2. Xarhgef3.2 Is Predominantly Expressed in the DMZ at the Gastrula Stage

**Figure 2:**
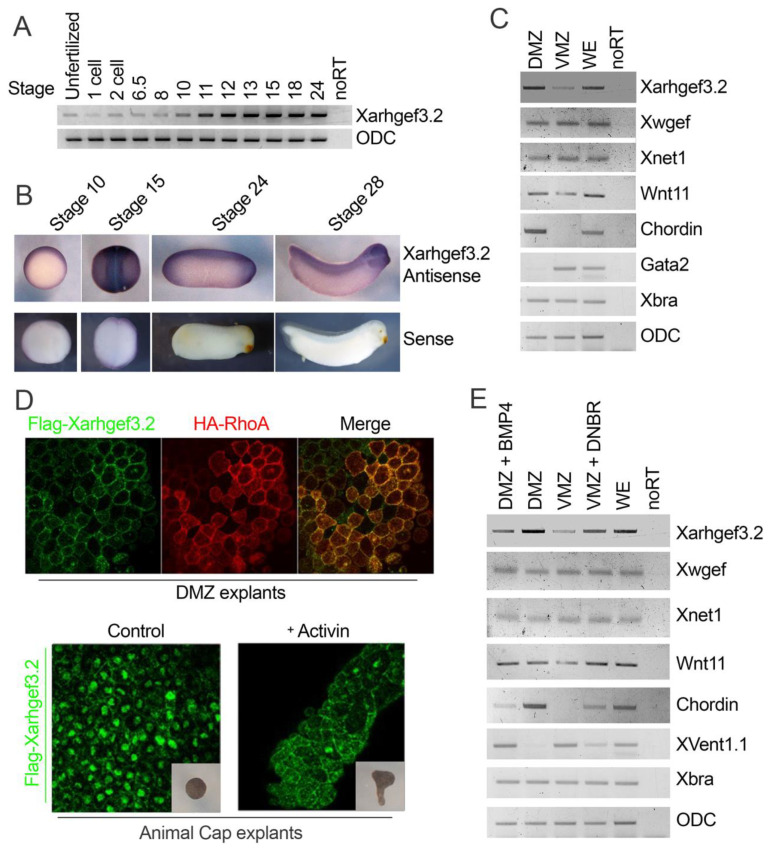
*Xarhgef3.2* is expressed in the DMZ at the gastrula stage. (**A**) Temporal expression pattern of *Xarhgef3.2* was analyzed using RT-PCR at various developmental stages as indicated. (**B**) Spatial expression of *Xarhgef3.2* was analyzed using whole mount in situ hybridization at the indicated stages. Stage 10: vegetal view, dorsal lip on top; stage 15: dorsal view; stages 20 and 28: lateral views. (**C**) RT-PCR confirmed the spatial expression pattern of the indicated gene expression by using each dissected (at stage 10) region of the embryo at stage 12. (**D**) RNAs of *Flag-Xarhgef3.2* (100 pg/embryo) and *HA-RhoA* (100 pg/embryo) were co-injected into the dorsal marginal zone at the four-cell stage. The DMZ explants were dissected at stage 10 and fixed using MEMFA and stained with the anti-Flag and anti-HA antibodies. Localization of Flag-Xarhgef3.2 was analyzed with the DMZ explants using confocal microscopy. (**E**) The BMP4- or DNBR-injected DMZ or VMZ explants were examined using RT-PCR to analyze the expression level of the indicated genes at stage 12 (ventral marker: *gata2* and *ventx1.1*; mesoderm marker: *xbra*; organizer marker: *chrd*; loading control: *odc*).

*Xarhgef3.2* was upregulated in the ACs by Bmp inhibition. During early normal gastrulation (around stage 10–11), Bmp is predominantly expressed in the VMZ and is inactivated in the DMZ [47,48]. To examine the inversed correlation between the Bmp gradient and the expression of *Xarhgef3.2*, both temporal and spatial expressions of *Xarhgef3.2* were analyzed by RT-PCR. First, the temporal expression of *Xarhgef3.2* was examined using RNA isolated from the whole embryos at successive developmental stages. Expression of *Xarhgef3.2* was seen starting from the unfertilized egg and registered to be significantly elevated from stage 10 (the stage when CE begins) while being maintained until the tadpole stage of stage 24 (Figure 2A). The temporal and spatial expression patterns of *Xarhgef3.2* were also determined using an in situ hybridization assay at different stages of *Xenopus* development. Previously, Hufton et al. reported that *Xarhgef3.2* is expressed around the dorsal marginal zone or the dorsal lip during the early gastrula stage embryo [49]. Similarly, we found that *Xarhgef3.2* expression is restricted in the dorsal organizer region. However, *Xarhgef3.2* expression gradually shifted to the neural fold region and the prospective head region at a later stage (neurula- and tadpole-stage *Xenopus*) (Figure 2B, stage 24 and 28). To confirm whether the Bmp4 gradient was inversely correlated with the endogenous expression pattern of *Xarhgef3.2*, VMZs and DMZs were dissected from the normal embryos at stage 10. To examine the normal dissection of the DMZ and the VMZ, the *chrd* expression of the DMZ, *gata2* of the VMZ, and *xbra* for pan-mesoderm specific markers were confirmed by RT-PCR (Figure 2C, lines 5–7). The expression of *Xarhgef3.2* was predominantly found in the DMZ and much less in the VMZ (Figure 2C, line 1; compare lanes 1 and 2). The results confirmed the data from the in situ hybridization assay; *Xarhgef3.2* was highly expressed in the dorsal region. On the other hand, previously, the expression levels of *Xnet1* and *Xwgef* [27,28] were shown to be similar in the dorsal and ventral regions (Figure 2C, lines 2 and 3). The similar expression levels of *net1* or *Xwgef* in the DMZ and the VMZ indicate that *Xarhgef3.2* is a specific candidate molecule, possibly modulated by the BMP gradient during the early onset of gastrula movement. The subcellular localization of *Xarhgef3.2* was further examined in the DMZ explants of the *Xenopus* embryos at stage 13. The characterized guanine nucleotide exchange factors such as Xwgef function with small GTPases in the plasma membrane [27]. Thus, cellular localization of Flag-tagged Xarhgef3.2 and HA-tagged RhoA was analyzed using immunostaining with an anti-Flag antibody. The results showed that Xarhgef3.2 was localized at the plasma membrane and colocalized with HA-tagged RhoA in the DMZ explants (Figure 2D). To rule out that the signal was not an autofluorescence artifact, the localization of Xarhgef3.2 was examined in the control AC and the activin-treated AC cases. As expected, Xarhgef3.2 was localized in the nucleus of the untreated control ACs (Figure 2D) and at the plasma membrane in the elongated ACs with activin treatment (Figure 2D). These results suggest that Xarhgef3.2 in the DMZ is functionally active as localized in the plasma membrane during CE cell movement of early gastrula embryos. We then examined whether the BMP gradient could indeed modulate the expression of *Xarhgef3.2* in the DMZ and the VMZ. According to our expectation, injection of bmp4 in the DMZ slightly reduced the expression of *Xarhgef3.2* (Figure 2E, line 1; compare lanes 1 and 2). On the other hand, injection of DNBR at the VMZ resulted in upregulated *arhgef3.2* expression (Figure 2E, line 1; compare lanes 3 and 4). In this experiment, some of the control markers including *chrd* (dorsal mesoderm, direct target of activin/Smad2,3 [50]), *gata2* (or *ventx1.1)* (ventral mesoderm and direct target of BMP/Smad1 [51]), *xbra* (pan-mesoderm, Fgf target [52]), and *wnt11* (*wnt11b*; noncanonical *wnt*) were shared as shown in Figure 1C to confirm gain and loss of function for BMP reversing the dorsoventral character of the cells while the mesoderm character was maintained. Taken together, the results suggest that Bmp gradient and the expression pattern of *Xarhgef3.2* are inversely correlated, and the *Xarhgef3.2* temporal expression pattern with its cellular localization in the DMZ also depicts an active function for Arhgef3.2 in gastrulation movement.

### 3.3. Overexpression of Xarhgef3.2 Modulates Gastrulation without Affecting the Cell Fate

**Figure 3:**
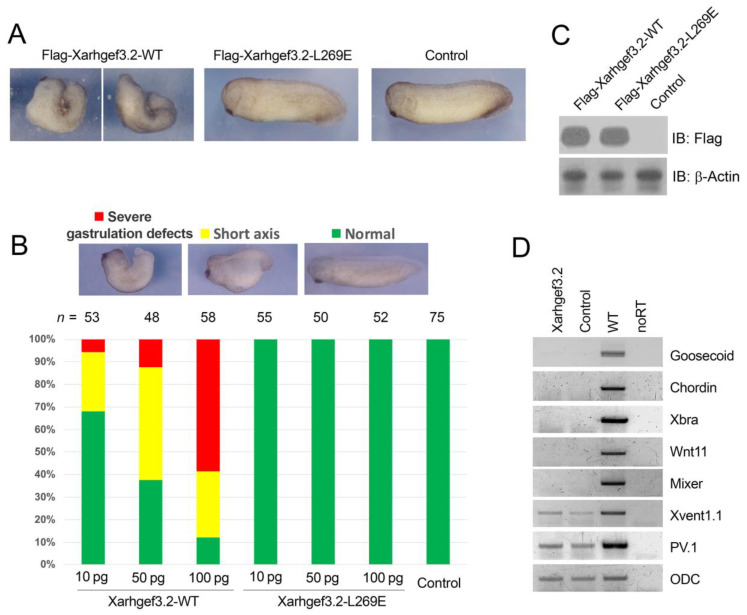
Overexpressed *Xarhgef3.2* induces gastrulation defects of *Xenopus* embryos. (**A**) RNAs of *WT-Xarhgef3.2* or *MT-Xarhgef3.2 (L269E)* were injected into the animal pole region at the one-cell stage (100 pg/embryo). (**B**) RNAs of *Flag-Xarhgef3.2* (100 pg/embryo) or *Flag-Xarhgef3.2 (L269E)* (100 pg/embryo) were injected at the one-cell stage. Phenotypes were evaluated as indicated at stage 28. (**C**) The expression level of each Flag-tagged construct was confirmed by Western blot analysis. (**D**) ACs were dissected from the *Xarhgef3.2* (100 pg/embryo)-injected embryos. The explants were incubated until stage 13, and RT-PCR was performed for analysis of the indicated gene expression.

To examine the functional role of Xarhgef3.2 in the early development of *Xenopus*, we performed gain-of-function assays in which the animal pole or dorsal blastomeres of the embryos were injected with mRNA encoding full-length *Xarhgef3.2* at the one-cell or four-cell stages. The embryos were observed for their morphological changes at stage 28. Ectopic expression of *Xarhgef3.2* in the animal pole region led to gastrulation defects in the whole embryos in a dose-dependent manner with the percentage of gastrulation-defective embryos increasing with a higher-dose injection of *Xarhgef3.2* mRNA (Figure 3). The embryos injected with *Xarhgef3.2* into each of the two dorsal blastomeres (25 pg/blastomere) at the four-cell stage resulted in remarkably reduced anterior structures with severely defective gastrulation (Figure 3A). The resultant morphologies implicate that overexpression of *Xarhgef3.2* may affect the cell movement of embryo gastrulation. To examine whether the gastrulation defect was caused by the GDP–GTP exchange activity of *Xarhgef3.2*, we cloned a mutant construct of *Flag-Xarhgef3.2 (L269E)* which loses the GDP–GTP exchange activity [53,54]. The gradually increased amounts of mRNA (10, 50, and 100 pg/embryo) of *wt (wild-type)-Flag-Xarhgef3.2* or *mt (mutant)-Flag-Xarhgef3.2 (L269E)* were injected into one-cell embryos and the phenotypic changes were analyzed at stage 28. The increased amount of the *WT-Xarhgef3.2* injection led to the proportionally increased gastrulation defects (Figure 3B: left-side graph). However, the ectopic expression of *Xarhgef3.2 (L269E)* (up to 100 pg/embryo) did not induce any gastrulation defects and hence suggested that the gastrulation defect caused by overexpression of *Xarhgef3.2* may depend on its GDP–GTP exchange activity (Figure 3B). To confirm protein expression of the injected mRNAs for the Flagged versions, the proteins were detected from the embryos using an anti-Flag antibody (Figure 3C). The similar experiment using *WT-Xarhgef3.2* and the *Xarhgef3.2 (L269E*) mutant was performed to rescue *Xarhgef3.2* knockout cells by You et al. (2021) [53]. We further eliminated the possibility that gastrulation might be impaired due to a change in gene expression. RT-PCR was performed to analyze gene expression using the ACs injected with *WT-Xarhgef3.2* mRNA. As shown in Figure 3D, overexpression of *Xarhgef3.2* did not change the expression patterns of dorsal mesodermal genes of the organizer for *goosecoid* (*gsc*) and *chrd*, the pan-mesodermal gene *xbra*, and the endodermal gene *mixer* expression in the ACs at stage 13. Collectively, these data emphasize that *Xarhgef3.2* overexpression impaired normal gastrulation whose defective function may depend on its GDP–GTP exchange activity without affecting cell fate determination.

### 3.4. Xarhgef3.2 Is Required for Normal Gastrulation of Xenopus Embryos

**Figure 4:**
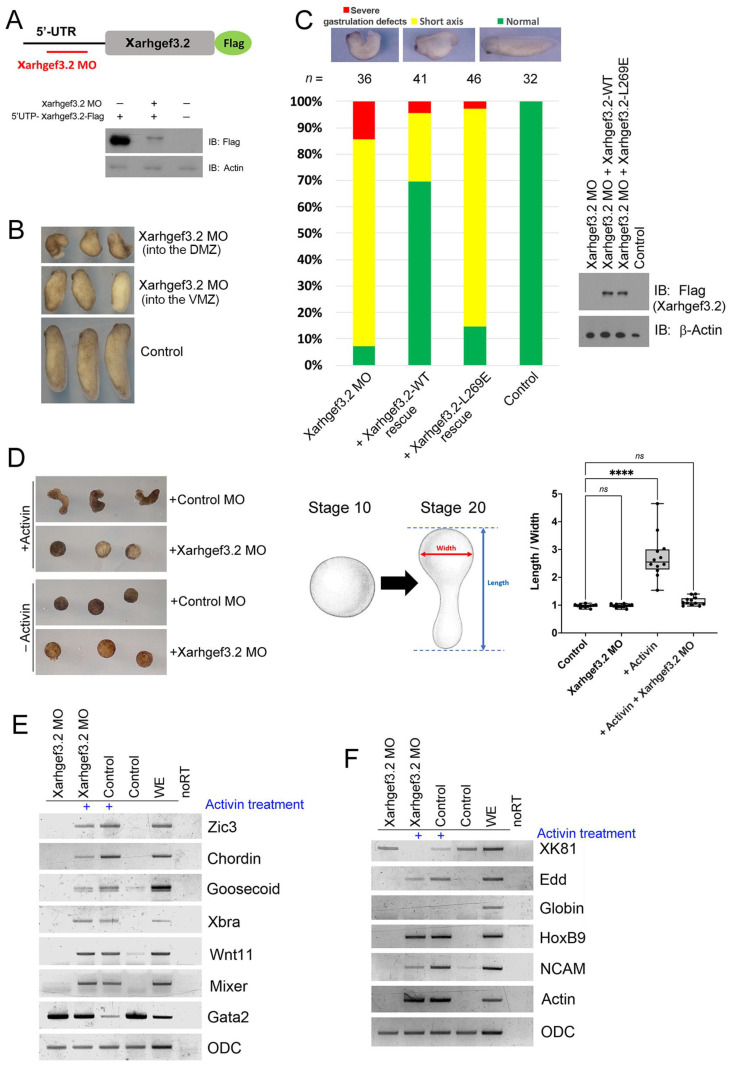
Knockdown of *Xarhgef3.2* leads to a gastrulation defect without affecting the cell fate. (**A**) The whole embryos were injected with RNAs of the *5′UTR-Xarhgef3.2-3Flag* construct (100 pg/embryo) along with the *Xarhgef3.2* MO (60 ng/embryo) as indicated. The embryos were used for Western blot check of specific knockdown of Xarhgef3. (**B**) The *Xarhgef3.2.L* MO was injected into the animal pole region at the one-cell stage (60 ng/embryo) or into two blastomeres of both the dorsal and ventral marginal zone at the four-cell stage (15 ng/blastomere). The morphological changes of the embryos were then examined at stage 28. (**C**) RNAs of *Flag-Xarhgef3.2* (50 pg/embryo; an off-target construct against the MO) or the *Xarhgef3.2* MO (60 ng/embryo) were injected to the one-cell stage embryos. Phenotypes were evaluated as indicated at stage 28. (**D**) The ACs injected with the MO of *Xarhgef3.2* were dissected at stage 8 and incubated with activin (50 ng/mL). Phenotypes of the ACs were evaluated at stage 16. The histogram depicts the width/length ratio of the animal cap explants. Quantification with one-way ANOVA, scatterplots represent the means ± SD from three biological repeats. Dunn’s multiple comparison, **** *p* < 0.0001, *ns*—no statistical differences between the groups. (**E**,**F**) The ACs injected with the MO of *Xarhgef3.2* were dissected at stage 8 and incubated in the presence or absence of activin (50 ng/mL) until stages 11 (**E**) and 24 (**F**). RT-PCR was performed for analysis of the indicated gene expressions.

To examine the functional requirement of *Xarhgef3.2*, a loss-of-function study was performed with antisense morpholino oligos targeting the endogenous *Xarhgef3.2* mRNA. Oligos target the 5′UTR region of *Xarhgef3.2* mRNA (Figure 4A). The inhibitory activity of the *Xarhgef3.2* morpholino oligo (MO) was evaluated with Western blot analysis by comparing the affected protein levels of the *5′UTR-Xarhgef3.2-3Flag* construct containing the 5′UTR region which includes the MO target sequence. In addition, 3Flag was tagged in the 3′ region for detection of the protein without protection of the MO action. The MO was confirmed to block translation of the target gene *5′UTR-Xarhgef3.2-3Flag* without affecting amounts of the endogenous *Xarhgef3.2* RNA (compare the first lane with the third lane of line 1 (stage 12) in Supplementary Figure S1C) and control protein actin as shown in Figure 4A (second line). Phenotypic changes were then observed by injecting the *Xarhgef3.2* MO. MO injection into the animal pole region at the one-cell stage (60 ng/embryo) led to axis shortening with a mild gastrulation defect in the whole embryos. Injection of the *Xarhgef3.2* MO into the marginal zone region of two dorsal blastomeres (15 ng/blastomere) at the four-cell stage resulted in an abnormal head structure and severe gastrulation defects (Figure 4B; dorsal marginal zone). However, when the same amount of the MO (15 ng/blastomere) was injected into the ventral marginal zone, no severe gastrulation defects were observed (Figure 4B; ventral marginal zone). The Control MO also did not cause any severe phenotypic changes in the whole embryos (Figure 4B; control). Differential severity of the gastrulation defect suggests that the endogenous function of *Xarhgef3.2* is required for normal gastrulation and a specific role in the dorsal mesoderm during gastrulation of *Xenopus* embryos. To confirm whether the gastrulation defect caused by the *Xarhgef3.2* MO specifically depended on injection of the MO, the morphological changes were compared in three different RNA-injected groups: (1) *Flag-Xarhgef3.2* alone, (2) *Xarhgef3.2* MO alone, and (3) co-injection of *Flag-Xarhgef3.2* and the MO together. The phenotypic changes were observed at stage 28. Co-injection with RNAs of *Flag-Xarhgef3.2* and the *Xarhgef3.2* MO (group 3) rescued the more severe gastrulation defects found either with *Flag-Xarhgef3.2* alone (group 1) or the *Xarhgef3.2* MO alone (group 2) (Figure 4C). Since the MO targeted the 5′-UTR region of *Xarhgef3.2*, a rescue experiment was performed using *5′Flag-Xarhgef3.2* which does not contain the 5′-UTR region of the *Xarhgef3.2* target sequence of the MO (compare the first lane with the second and third lanes of protein bands in Figure 4C). The results indicate that proper activity of Xarhgef3.2 is necessary for normal CE movement of gastrula embryos. We then performed AC elongation assays using the *Xarhgef3.2* MO. Activin treatment can instigate elongation accompanying active cell migration and CE movement in the AC system of *Xenopus* embryos [44,55]. *Xarhgef3.2* MO injection resulted in effective reduction of the elongation found in the activin (50 ng/mL)-treated ACs (Figure 4D). Although certain changes in the markers were observable, we repeatedly found that the cell fate was not affected much by the *Xarhgef3.2* MO which was confirmed by RT-PCR at stage 11 and stage 24 (Figure 4E, stage 11, and Figure 4F, stage 24). The results of the AC assay further support the view that Xarhgef3.2 is necessary for CE of *Xenopus* embryos. Together, loss-of-function studies for *Xarhgef3.2* suggested that Xarhgef3.2 is an essential molecule necessary for gastrulation cell movement which is functionally more active in the DMZ of gastrula embryos.

### 3.5. Xarhgef3.2 Specifically Interacts with RhoA and Regulates CE Cell Movement through Modulation of Noncanonical Wnt Signaling

**Figure 5:**
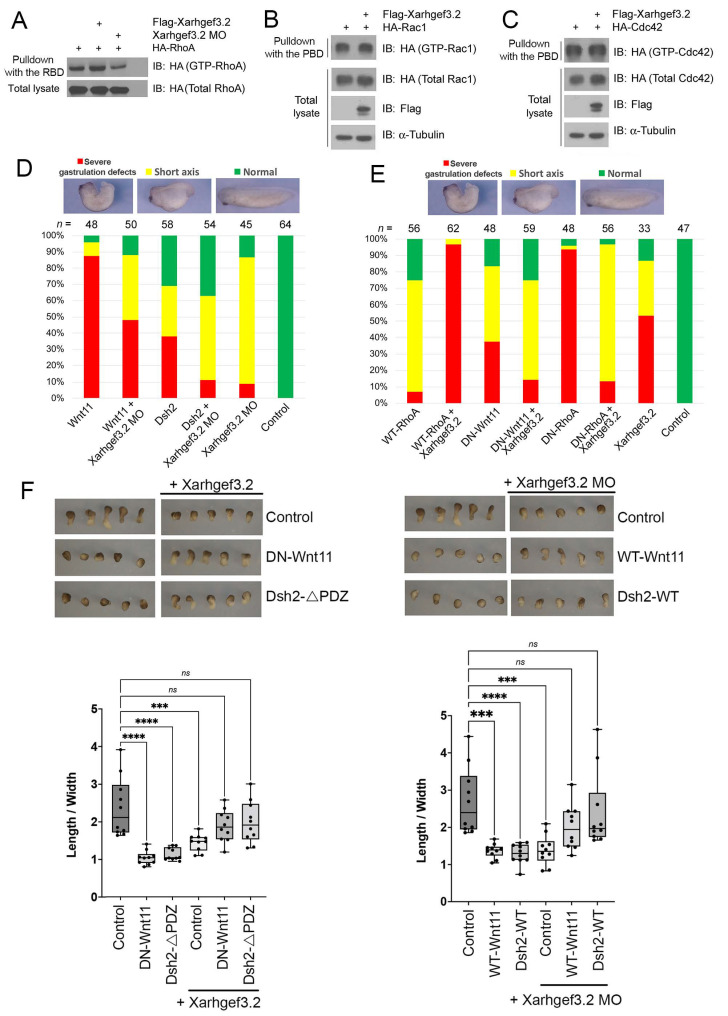
Xarhgef3.2 regulates CE movement in a noncanonical Wnt signal cascade. (**A**) The DMZ explants injected with *Xarhgef3.2* into the dorsal marginal zone at the four-cell stage (25 pg/blastomere) or the *Xarhgef3.2* MO (15 ng/blastomere) were dissected at stage 10. Rho-binding domain (rhotekin, RBD) pulldown assay was performed for the analysis of RhoA activity. (**B**,**C**) The DMZ explants injected with *Xarhgef3.2* into the dorsal marginal zone at the four-cell stage (25 pg/blastomere) were dissected at stage 10. The Rac1- or Cdc42 (p21)-binding domain (PAK1, PBD) pulldown assay was performed for the analysis of Rac1 or Cdc42 activity. (**D**) WT-Wnt11 (1 ng) or WT-Dsh2 (1 ng) RNAs were injected singly or co-injected with the *Xarhgef3.2* MO (60 ng) into the animal pole region at the one-cell stage. Phenotypes were evaluated as indicated at stage 28. (**E**) *WT-RhoA* (500 pg), *DN-wnt11* (1 ng), and *DN-RhoA* (500 pg) RNAs were injected singly or co-injected with the *Xarhgef3.2* RNA (100 pg) into the animal pole region at the one-cell stage embryos. Phenotypes were evaluated as indicated at stage 28. (**F**) The DMZ explants injected with RNAs and the *Xarhgef3.2* MO as indicated were dissected at stage 10 and incubated until stage 16. The histogram depicts the width/length ratio of the Keller explants. Quantification with one-way ANOVA, scatterplots represent the means ± SD from three biological repeats. Dunn’s multiple comparison, *** *p* < 0.001, **** *p* < 0.0001, *ns*—no statistical differences between the groups.

Gastrulation movement of *Xenopus* embryos is explicitly regulated by noncanonical Wnt-PCP signaling [56] and RhoA functions at the downstream target of this pathway [24]. As shown by subcellular localization and gain-of-function assays of Xarhgef3.2 with the affected CE cell movement of the dorsal marginal zone, we assume that Xarhgef3.2 functions as a component of the Wnt-PCP signal cascade and interacts with RhoA. Having insight into the specificity of Rho family GEFs is important for understanding the biological roles of these proteins; we thus examined whether this interaction was limited to RhoA or if there were other small GTP-binding proteins of the Wnt-PCP pathway, Rac1 and Cdc42, that were also involved. A prolonged activation of RhoA via a constitutively active mutant of RhoA leads to gastrulation defects [22,45], and previous studies reported that human Arhgef3 activates RhoA only but not Rac1 and Cdc42 [30]. We examined the specificity of Xarhgef3.2 by carrying out an RBD (Rho-binding domain) pulldown assay using the DMZ explants injected with the *Xarhgef3.2* mRNA or *Xarhgef3.2* MO. Since RBD is known to bind only to GTP-bound active RhoA [29], the band intensity of the RBD-bound RhoA is proportional to the amount of active RhoA. Ectopic expression of *Xarhgef3.2* enhanced RhoA activity, and injection of the *Xarhgef3.2* MO reduced the RhoA activity (Figure 5A). In contrast, the Rac1 or Cdc42 (p21)-binding domain (PBD) pulldown assay demonstrated that the activities of Rac1 or Cdc42 were not changed by overexpression or knockdown of *Xarhgef3.2* (Figure 5B). Thus, we conclude the interaction of *Xenopus* Arhgef3.2 is limited only to RhoA.

We also performed an epistatic analysis with Wnt-PCP components, Wnt 11, Dsh2, that are known to modulate noncanonical Wnt signaling in order to further validate our assumptions. Ectopic expression of WT-Wnt11 or WT-Dsh2 induced gastrulation defects in the whole embryos, but co-injection with the *Xarhgef3.2* MO rescued the phenotypic defects (Figure 5D). Gastrulation defects caused by injection with the mRNA of *RhoA* was significantly increased with co-injection with the *Xarhgef3.2* RNA. The whole embryos injected with the mRNAs of *DN-wnt11* or *DN-RhoA* also resulted in gastrulation defects, but co-injection with the mRNAs of *Xarhgef3.2* decreased the phenotypic defects (Figure 5E). Our hypothesis was also substantiated using Keller explants. Ectopic expression of WT-Wnt11, DN-Wnt11, or WT-Dsh2 caused a reduction of elongation in the Keller explants compared to the control. However, co-injection with the mRNAs of *Xarhgef3.2* or the *Xarhgef3.2* MO rescued the reduced elongation (Figure 5F, also see the Keller explant pictures and the statistical measurement data of elongation). Together, these data suggest that Xarhgef3.2 may function as a regulatory component downstream of Wnt-PCP signaling and controls CE movement by specifically activating RhoA.

### 3.6. N-Terminal Region of Xarhgef3.2 Interacts with Dsh2 through Daam1 under the Wnt-PCP Signaling Stimulation

**Figure 6:**
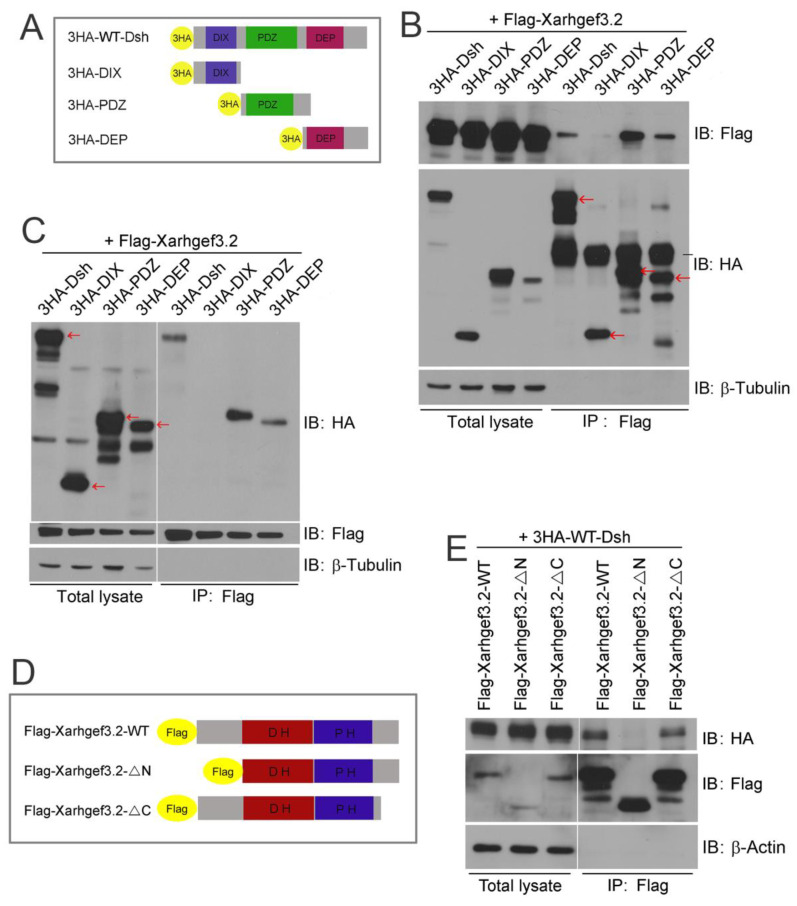
N-terminal region of Xarhgef3.2 is required for interaction with the PDZ and the DEP domain of Dsh2. (**A**) Schematic representation of HA-tagged constructs for Dsh2. (**B**,**C**) RNAs of *Flag-Xarhgef3.2* (100 pg) were co-injected with *HA-WT-Dsh2* (1 ng), HA-PDZ (1 ng), or HA-DEP (1 ng) at the one-cell stage. Immunoprecipitation assays were performed with an anti-Flag antibody or an anti-HA antibody at stage 10. The red arrows indicate intact protein bands. (**D**) Schematic representation of Flag-tagged constructs of *Xarhgef3.2*. (**E**) RNAs of *HA-WT-Dsh2* (1 ng) were co-injected with *Flag-WT-Xarhgef3.2*, *Flag-ρN- Xarhgef3.2*, or *Flag-ρC-Xarhgef3.2* (each, 100 pg) at the one-cell stage. Immunoprecipitation assays were performed with an anti-HA antibody at stage 10.

**Figure 7:**
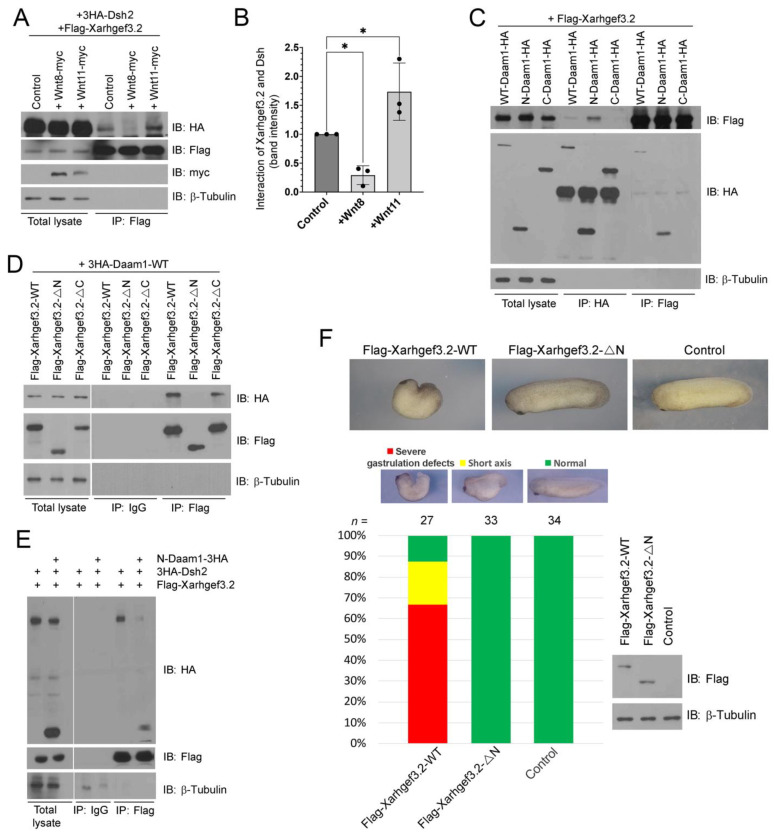
Xarhgef3.2 regulates gastrulation cell movements through interacting with noncanonical Wnt signaling components. (**A**) RNAs of *Flag-Xarhgef3.2* (100 pg) and *HA-Dsh2* (1 ng) were co-injected with *wnt8-myc* (500 pg) or *wnt11-myc* (500 pg) at the one-cell stage. Immunoprecipitation assay was performed with an anti-Flag antibody at stage 10. (**B**) The graph indicates the relative interaction level between Flag-Xarhgef3.2 and HA-Dsh2. Quantification with one-way ANOVA, scatterplots with bar represent the means ± SD from three biological repeats. Dunn’s multiple comparison, * *p* < 0.05. (**C**) RNAs of *Flag-Xarhgef3.2* (100 pg) were co-injected with *HA-WT-daam1*, *HA-N-daam1*, or *HA-c-daam1* (each, 1 ng) at the one-cell stage. Immunoprecipitation was performed with an anti-Flag antibody or an anti-HA antibody at stage 10. (**D**) RNAs of *HA-WT-daam1* (1 ng) were co-injected with *Flag-WT-Xarhgef3.2*, *Flag-ρN-Xarhgef3.2*, or *Flag-ρC-Xarhgef3.2* (each, 100 pg) at the one-cell stage. Immunoprecipitation assay was performed with an anti-HA antibody at stage 10. (**E**) RNAs of *Flag-Xarhgef3.2* (100 pg) and *HA-Dsh2* (1 ng) were injected or co-injected with *HA-N-daam1* (2 ng) at the one-cell stage. Immunoprecipitation was performed with an anti-Flag antibody at stage 10. (**F**) RNAs of *Flag-Xarhgef3.2* (100 pg) or *Flag-ρC-Xarhgef3.2* (100 pg) were injected at the one-cell stage. Phenotypes were evaluated as indicated at stage 28. The expression level of each construct was confirmed by Western blot analysis.

Our in vivo experiments illustrate that Xarhgef3.2 functions in the induction of RhoA in response to Wnt signaling. RhoA acts as a downstream target of noncanonical Wnt signaling by associating with Disheveled (XDsh2), and this association is interceded by Daam1 [24]. Our data showed that Xarhgef3.2 may act as a downstream component of noncanonical Wnt signaling in the DMZ. To gain more information about the mechanism of Xarhgef3.2 function, we performed biochemical studies with Dsh2 and Daam1, known vital components of noncanonical Wnt signaling [24,57]. We generated various mutant constructs containing each domain of Dsh2 as indicated in Figure 6A. Then, coimmunoprecipitation assays were performed between Xarhgef3.2 and Dsh2, and they showed that the PDZ and DEP domains of Dsh2 interact with Xarhgef3.2 (Figure 6B,C). To identify the portion of Xarhgef3.2 interacting with Dsh2, we performed coimmunoprecipitation assays with deletion mutants of Xarhgef3.2 as indicated (Figure 6D). The results show that the N-terminal of Xarhgef3.2 is bound to Dsh2 (Figure 6E). In addition, IP with serial deletion mutants of Xarhgef3.2 showed that the most N-terminal region of Xarhgef3.2 is required for the interaction with Dsh2 (Supplementary Figure S3A,B). We also showed that the nuclear localization signal (NLS) deletion mutants (*ρ*N3) were predominantly localized at the plasma membrane. However, the Xarhgef3.2-*ρ*N3 mutant did not interact with Dsh2. (Supplementary Figure S3B,C). We then investigated whether the interaction between Xarhgef3.2 and Dsh2 increased under upregulation of Wnt-PCP signaling. Ectopic expression of *Wnt11* increased binding between Xarhgef3.2 and Dsh2, but not with *wnt8*, a typical form of canonical Wnt, as it decreased this interaction (Figure 7A,B). Furthermore, we examined the interaction between Xarhgef3.2 and Daam1 as Daam1 binds to the PDZ and the DEP domain of Dsh2 [24], and the Xwgef N-terminal region interacts with Daam1 [27]. We found Xarhgef3.2 was immunoprecipitated with WT-Daam1 or N-Daam1 through the N-terminal part of Xarhgef3.2 (Figure 7C,D). Interaction between Xarhgef3.2 and Dsh2 in the presence of excessive N-Daam1 was analyzed to see whether the interaction of Xarhgef3.2 with Dsh2 was mediated by Daam1. Overexpression of N-Daam1 reduced the interaction between Xarhgef3.2 and Dsh2 (Figure 7E). These results suggested that Xarhgef3.2 may interact with Dsh2 through Daam1. We also examined colocalization of Xarhgef3.2 and Dsh2 or Xarhgef3.2 and Daam1. As shown, Xarhgef3.2 colocalized with Dsh2 and Daam1 at the plasma membrane in the DMZ explants (Supplementary Figure S4).

We next describe the physical interaction of Xarhgef3.2 with Dsh2 and Daam1 by observing phenotypic changes in the whole embryos injected with the RNAs for *Flag-WT-Xarhgef3.2* or Flag-*ρ*N-*Xarhgef3.2* (Figure 6D; N-terminal domain (including the NLS domain) deleted construct), with products that do not bind with Daam1. Overexpressing *Flag-ρN-Xarhgef3.2* produced a phenotype similar to the phenotype observed in the control whole embryos (Figure 7F). Our results collectively point that in the presence of Wnt ligand signaling, Xarhgef3.2 physically interacts with Dsh2 via Daam1 by means of its N-terminal region and activates downstream RhoA which is necessary for CE cell movement in *Xenopus* development.

## 4. Discussion

In the present study, we hypothesized that cell fate determination of germ layers and subsequent gastrulation movement are linked. In this perspective, we aimed to uncover the pathway links between these two events. We focused on the possible interaction of the Wnt-PCP component with the established Bmp gradient early gastrula of *Xenopus* embryos. In the current study, we reasoned that Bmp gradient inversely regulates gastrulation cell movement and found that transcription of *Xarhgef3.2* was regulated by the Bmp gradient. Altogether, the present study may add clues to embryonic morphogenesis in which cell fate determination of germ layers and subsequent gastrulation movement may be linked via transcriptional modulation of small GTPase regulators which play roles in the regulation of all the morphogenetic movements of developing gastrula embryos.

### 4.1. BMP Gradient Inversely Correlates with CE Movement

In this study, we first inquired about the involvement of the Bmp gradient as linking germ layer determination and gastrulation. The reasoning was based on the following: first, the spatial expression and activity pattern of Bmp are known, and Bmp signal gradient is high in the ventral side and low in the dorsal side of an embryo [48]. In contrast, in early gastrulation, CE cell movement is almost absent in the ventral side and highly present in the dorsal lip region where the Bmp gradient is at a minimum [58]. The spatial patterns of Bmp activity and CE activity are inversely correlated. Second, the temporal involvement of Bmp is compelling in that Bmp is a morphogen which actively participates in germ layer determination before gastrulation [47,48] as well as dorsoventral patterning of the mesoderm and the ectoderm (neuroectoderm vs. ectoderm) before and during gastrulation [59]. Bmp gradient has a role in the timely onset of gastrulation. Therefore, it is possible that Bmp gradient before gastrulation may prepare for subsequent CE movement of gastrulation. Third, according to the loss- and gain-of-function studies of Bmp, depletion of Bmp in the ventral side leads to formation of a secondary axis, whereas ectopic expression of Bmp in the dorsal region results in a ventralized embryo being headless with axis shortening [34]. Although whether secondary axis formation caused by depletion of Bmp requires active CE movement needs to be examined, the results indicate that the establishment of CE cell movement is dependent on Bmp gradient. Details of how Bmp gradient affects cell movement without affecting fate determination need to be understood. Fourth, there was an elongation study of ACs performed by Suzuki et al. [44] where co-injection of *dnbr* dramatically increased the elongation caused by the injection of *activin* and fgf in ACs. Since elongation is followed by the morphogenetic movement of involuting cells of the dorsal marginal zone during gastrulation [60], this elongation via Bmp inhibition strongly advocates for its correlation with gastrulation movement. In addition, it has been reported that Bmp gradient has an instructive role in establishing a reverse gradient of cell–cell adhesiveness to determine the direction of lateral mesodermal cell migration during dorsal convergence in zebrafish gastrula [61]. A repulsive role for Bmp gradient on lateral migrating mesodermal cells via diminishing cell–cell adhesion which is independent of fate specification and noncanonical Wnt signaling has also been reported [61]. Even though an interconnection between the Bmp gradient and gastrulation movement in gastrula embryos seems plausible, the studies of the involvement of the Bmp gradient in CE are limited, and more research is needed to support the conjecture that Bmp gradient regulates cell movement independently from cell fate specification.

We hypothesized Bmp as a key linking molecule between early primordial three germ layers formation and subsequent gastrulation movement, leading to having each germ layer localized to a specific region in a whole embryo. In this respect, we wonder whether the partial secondary axis formed by inhibition of the Bmp signal in the ventral side requires active CE movement. In our experiments, the secondary axis formed by the DNBR injection was abolished by DN-RhoA co-injection (Figure 1B,C), indicating that secondary axis formation also requires activation of small GTPases RhoA, possibly as a component of the Wnt-PCP pathway of CE movement. This Wnt-PCP activation for secondary axis formation in a whole embryo and enhanced elongation in ACs [44] and VMZ explants (Supplementary Figure S1B) could then be modulated in various ways, including signal cross-talks via post-translational modification, transcriptional regulation of a Wnt-PCP component, or both under the condition of BMP inhibition. Bmp inhibition in the ventral mesoderm converts the ventral mesoderm to the dorsal mesoderm which contains the organizer character [62]. Organizer transplantation to the ventral side of another embryo results in formation of a duplicated complete axis [63]. Secondary axis formation may require the dorsal mesoderm as the leading cells (bottle cells) as well as activation of Wnt-PCP signaling for proper gastrulation movement. From this set of observations, in the current study, we focused on the identification of the responsible regulators of small GTPases possibly involved in the Wnt-PCP pathway, whose expression would be upregulated under a BMP signaling block such as ectopic DNBR expression.

### 4.2. Bmp Inhibition Upregulates Expression of a Regulator of Small GTPases, Xarhgef3.2

Bmp depletion converts the ventral mesoderm to the dorsal mesoderm and leads to elongation of VMZ explants (Supplementary Figure S1B). We next examined the possibility that Bmp inhibition may upregulate expression of the genes related to cell movement of gastrula embryos. Bmp inhibition, activin or Fgf treatment in the ACs resulted in formation of different germ layers, namely neuroectoderm, organizer (dorsal mesoderm), and lateral mesoderm, respectively. Activin has been shown to transform ectodermal explant cells to three different fates of the dorsal mesoderm, endoderm and neuroectoderm along with elongation in ACs. Fgf also leads to mesoderm formation of ACs with elongation being a little less than that of activin. Meantime, DNBR induces neuroectoderm formation in ACs but DNBR alone results in minor elongation of ACs compared to that of activin and Fgf. Interestingly, combined treatment of DNBR with activin or Fgf results in much enhanced elongation [44], which shows that Bmp signaling in combination with activin or Fgf has an additional role in elongation. The elongation of ACs could possibly be affected by CE movement and Wnt-PCP activation by modulating the activity or the expression of Wnt-PCP component molecules. Since all the three conditions of activin, Fgf, and Bmp inhibition commonly lead to elongation accompanying fate change in ACs, this elongation induction character was used to identify any modulators involved in cell movement of gastrula embryos, including modulators of the Wnt-PCP component gene whose expression would be increased in all the three conditions. We performed a microarray analysis using RNA from DNBR-injected, activin- or Fgf-treated ACs. *Xarhgef3.2.L* was found to become commonly upregulated in all the three instances (Figure 1D).

In the current study, we mainly focused on characterization of *Xarhgef3.2* whose transcription was upregulated when Bmp signaling was inhibited. Besides *Xarhgef3.2*, it would be interesting, regulated under BMP inhibition/stimulation conditions, to screen for additional components of the stimulatory/inhibitory Wnt-PCP pathway, respectively. In zebrafish, it has been observed that a single gradient of Bmp activity specifies the cell fate as well as regulates the process of CE by negatively modulating expression of the noncanonical Wnt ligands, wnt11 and wnt5a [10]. In addition, a dorsoventral gradient of *wnt11b* was detected at stage 11 by Tada and Smith, 2000 [13], and also in the more recent transcriptome analysis of dorsal and ventral halves at stage 10.5 in *Xenopus* by Ding et al., 2017 [64]. In the present study, however, *wnt11b* expressions were not very different in the DMZ and VMZ explants at stage 12 (Figure 2C), and *wnt11b* expression was not significantly modulated by Bmp overexpression in the DMZ or BMP inhibition in the VMZ according to the RT-PCR analysis (Figure 2E). This suggested that *wnt11b* expression needs to be confirmed in more stages and with better quantification in early *Xenopus* gastrula.

The increased expression of *Xarhgef3.2* was reported by Hufton et al. (2005) [49] and Popov et al. (2017) [65]. Hufton et al. screened for *Xarhgef3.2* using the same Affimatrix oligonucleotide microarray as in our study. *Xarhgef3.2* was upregulated at stage 10.5 by joint ectopic overexpression of *nog* and *dkk-1* cDNA, respective inhibitors of the BMP and Wnt pathways in the VMZ. *Xarhgef3.2* was not expressed with ectopic overexpression *nog* or *dkk-1* alone. In the present study, *Xarhgef3.2* levels were detected under the BMP inhibition condition and without the Wnt inhibition in both the VMZ and AC explants. In comparison, we used the VMZ explants injected with DNBR mRNA and Hufton et al. used cDNA to ensure that the molecules were only expressed after the start of zygotic transcription (mid-blastula transition), thereby mimicking the endogenous regulation of these genes. In addition, they observed *arhgef3.2* expressions via in situ analysis using whole embryos, and we used the VMZ explants. At the present time, we do not know the exact reason how the two experiments lead to the *Xarhgef3.2* expression differences observed. More recently, Popov et al. also reported *arhgef3* as one of the regulators which are differentially enriched in the DMZ of *Xenopus* at stage 11 [65]. Any roles for activin, Fgf, and Bmp gradient changes in modulation of the target molecule such as its post-translational modification for its activation and deactivation in the Wnt-PCP component pathway do merit a closer look. Regarding *Xarhgef3.2*, it also remains unclear whether its transcriptional upregulation was mainly due to Bmp signal inhibition or it was also independently mediated by Fgf and activin signaling. We need to remember that there is an inhibitory property of Fgf signaling on the Bmp pathway through Bmp intracellular signal mediator Smad1 link region phosphorylation [66]. Similarly, there is also the antagonistic behavior of activin signaling on Bmp signaling through induction of BMP antagonists such as *chrd*, *nog*, and *cerberus* [67]. We may assume that upregulation of Wnt-PCP component *Xarhgef3.2* via Bmp inhibition is at least partially common. In addition, the fact that combination treatment with DNBR/activin or DNBR/Fgf results in a significantly more enhanced elongation [44] (our unpublished data) provides for the possibility of additional modes of Wnt-PCP component Xarhgef3.2 being regulated, such as its post-translational modification or regulation of its intracellular localization.

### 4.3. Xarhgef3.2 Is a Component of Wnt-PCP Signaling without Affecting Fate Determination

**Figure 8:**
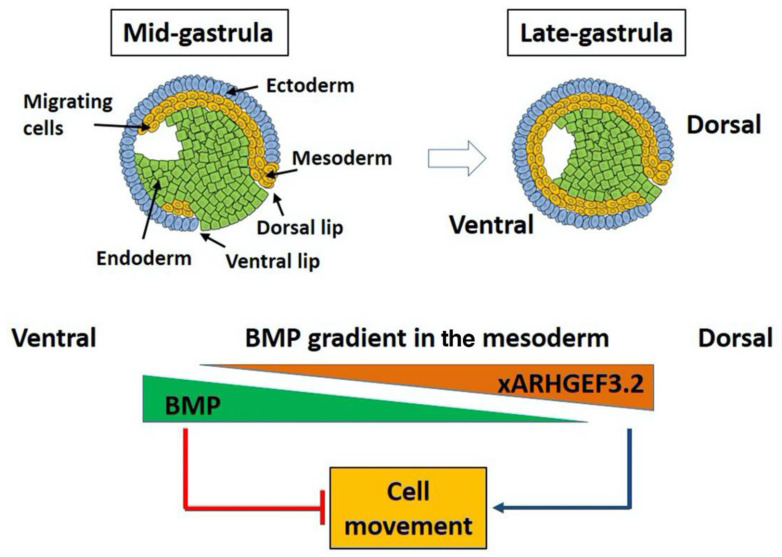
Proposed model of the Bmp-Arhgef3.2 gradient in the modulation of cell movement in the gastrula *Xenopus* embryos. In the proposed model, Bmp gradient inversely modulates expression of *Xarhgef3.2*, which in turn regulates cell movement in gastrula embryos, leading to active gastrulation movement in the dorsal region and a weak gastrulation movement in the ventral region of the embryos.

We found that *Xarhgef3.2* is highly expressed in the DMZ and less so in the VMZ (Figure 2C). In the present study, we tried to provide evidence on Xarhgef3.2 as a candidate component of Wnt-PCP signaling involved in CE movement. Our claim of CE and Wnt-PCP being linked with Arhgef3.2 functioning are supported by the following: first, the function of another tissue-specific RhoGEF protein, plekhg5, has been reported in apical constriction of bottle cells of gastrulation in *Xenopus* embryos. However, apical constriction activity of bottle cells is not shared by *arhgef3* [68]. Second, Xarhgef3.2 binds to a noncanonical Wnt-PCP component, Daam1 (Figure 7D). Third, Arhgef3.2 binding with Dsh2 is enhanced by noncanonical Wnt11 but not by canonical Wnt8 (Figure 7A). Fourth, both AC elongation from activin treatment and DMZ explant elongation are abolished by the *Xarhgef3.2* MO without obviously affecting gene expression patterns (Figure 4D–F and Figure 5F). Fifth, the reduced elongation of DMZ explants via activation or deactivation of each Wnt-PCP component alone is rescued by co-injection with the *Xarhgef3.2* partner with an opposite activity (WT-*Xarhgef3.2* MO vs. *Xarhgef3.2*), respectively (Figure 5F). The expression pattern of *Xarhgef3.2* is distinct from those of other well-characterized RhoGEFs (Net and Wgef) in *Xenopus*. The expression levels of the known RhoGEFs, *Xnet* and *Xwgef*, are similar in the DMZ and the VMZ (Figure 2C). This suggests that Xarhgef3.2 is a crucial component of Wnt-PCP that is functionally involved in CE movements and might be linked to BMP signal gradient. As summarized above, we provide multiple pieces of evidence to decipher the role of Xarhgef3.2 in noncanonical Wnt-PCP signaling and how it efficiently regulates CE. First, *Xarhgef3.2* is mainly expressed in the DMZ where CE is highly active and colocalizes in the membrane with RhoA (Figure 2). Xarhgef3.2 also modulates gastrulation without affecting the cell fate (Figure 3A,D and Figure 4B,D,E), and it specifically activates RhoA (Figure 5). N-terminal region of Xarhgef3.2 interacts with downstream Wnt signal mediators, Dsh2 and Daam1 (Figure 6 and Figure 7). The results of RhoA-specific activation by Xarhgef3.2 are reproducible, also seen in a previous report of human Arhgef3 [31]. A previous observation of *Xenopus* Wgef binding to Daam1 through its N-terminal domain [30] is also similar to our results with respect to Xarhgef3.2 interacting with Daam1 (Figure 7C,D). In the present study, we emphasized that Xarhgef3.2 is a component of Wnt-PCP signaling and its expression is modulated by the Bmp gradient. Although we tried to provide some evidence that Xarhgef3.2 is a functional component of noncanonical Wnt-PCP signaling involved in CE movement of gastrula embryos, the limitation of the present work remained to clarify whether other movements are also affected by Xarhgef3.2 during the gastrulation movement. In summary, we propose a model for Bmp gradient modulation of Xarhgef3.2 gradient via modulation of its expression, which in turn regulates cell movement in gastrula embryos (Figure 8). Human Arhgef3 has been linked with osteoporosis [69] and has oncogenic correlation with nasopharyngeal carcinoma (NPC), where a gain of function results in NPC tumorigenesis and metastasis and loss of function dramatically induces apoptosis in cancer cells [70]. As human and *Xenopus* Arhgef3.2 display high sequence homology, it will be interesting to see whether Bmp has a transcriptional regulatory role for human Arhgef3 as well and whether in human cells activin/TGF-beta/Fgf signaling modifies its physiological functions, leading us to define a common mechanism in understanding its role in cancer cells as well. Altogether, our study may provide an additional insight on the role of Bmp gradient on gastrula cell movement in addition to its known function in germ layer specification of vertebrate embryogenesis.

## Figures and Tables

**Table 1:** Primers used for RT-PCR amplification.

Gene Name	Sequence (5′ to 3′)	References
Actin	F-GCTGACAGAATGCAGAAG	[36]
	R-TTGCTTGGAGGAGTGTGT	
Chordin	F-TTAGAGAGGAGAGCAACTCGGGCAAT	[39]
	R-GTGCTCCTGTTGCGAAACTCTACAGA	
Edd	F-CTCGCTCTGGACAAAACTC	[36]
	R-GAGCTTCTTGATGGGAATG	
Gata2	F-AGGAACTTTCCAGGTGCATGCAGGAG	[36]
	R-CCGAGGTGCAAATTATTATGTTAC	
Globin	F-CATGGCTCTGCTGATCTGCCAACCAC	[36]
	R-CCCAGGCTGGTGAGCTGCCCTTGCTG	
Gsc	F-GCTGATTCCACCAGTGCCTCACCAG	[39]
	R-GGTCCTGTGCCTCCTCCTCCTCCTG	
Hoxb9	F-TACTTACGGGCTTGGCTGGA	[36]
	R-AGCGTGTAACCAGTTGGCTG	
Mixer	F-CACCAGCCCAGCACTTAACC	[36]
	R-CAATGTCACATCAACTGAAG	
Ncam	F-CACAGTTCCACCAAATGC	[36]
	R-GGAATCAAGCGGTACAGA	
XVent1.1 (Ventx1.2)	F-TTCCCTTCAGCATGGTTCAAC	[40]
	R-GCATCTCCTTGGCATATTTGG	
PV.1 (Ventx1.1)	F-CCTTCAGCATGGTTCAACAG	[40]
	R-CATCCTTCTTCCTTGGCATC	
Wnt11 (Wnt11b)	F-TGACAGCTGCAACCTCATGT	Current study
	R-ACAGAGGGCTGTCAGTGCTT	
Xarhgef3.2	F-ACCTCTCTCAAGAGTCACATCAC	Current study
	R-TACAGTAGCTGTCGTAGGAGTTC	
Xbra	F-GGATCGTTATCACCTCTG	[36]
	R-GTGTAGTCTGTAGCAGCA	
Xk81	F-TGGTGTTGAACAAGTGCAGG	[41]
	R-ACCTCCTCGACAATGGTCTT	
Xnet1	F-GACAAATTGGAGTACCTC	[28]
	R-CACCAAAGTCTCTTTTTTCTGCGG	
Xwgef	F-GAGGTGCCGGGGGAGGTTTTC	[27]
	R-GGGGGCCCGTCGCTGTAGTT	
Zic3	F-TCTCAGGATCTGAACACCT	[36]
	R-CCCTATAAGACAAGGAATAC	
ODC	F-GTCAATGATGGAGTGTATGGATC	[39]
	R-TCCATTCCGCTCTCCTGAGCAC	

## Data Availability

Original data are available on reasonable request from the corresponding authors.

## Supplementary Materials

The following supporting information can be downloaded at https://www.mdpi.com/article/10.3390/cells11010044/s1. Figure S1: Dominant negative form of RhoA (DN-RhoA) suppressed the secondary axis formation induced by DNBR. Figure S2: Alignment of Xenopus arhgef3.2 sequences with human, chick, lizard, zebrafish and mouse ARHGEF3 sequences. Figure S3: Truncated nuclear localization signal (NLS) block Arhgef3.2 nuclear localization. Figure S4: Arhgef3 is co-localized with Dsh and Daam-1. Table S1: Normalized and analyzed microarray data of upregulated genes from activin treatment. Table S2: Normalized and analyzed microarray data of upregulated genes from Dnbr treatment. Table S3: Normalized and analyzed microarray data of upregulated genes from Fgf treatment.
