# Supplementary material for: Bmp Signal Gradient Modulates Convergent Cell Movement via Xarhgef3.2 during Gastrulation of Xenopus Embryos
Source: Cells. 2021 Dec 24;11(1):44. doi: 10.3390/cells11010044 (PMC8750265; doi:10.3390/cells11010044)
Supplement: Supplementary file 1 [file cells-11-00044-s001.zip › cells-1447007-Phylogenetic tree related information.pdf]

**Protein sequence (FASTA) and information**

>sp|Q9NR81|ARHG3_HUMAN Rho guanine nucleotide exchange factor 3 OS=Homo sapiens OX=9606 GN=ARHGEF3 PE=1 SV=1
MVAKDYPFYLTVKRANCSLELPPASGPAKDAEEPSNKRVKPLSRVTSLANLIPPVKATPL
KRFSQTLQRSISFRSESRPDILAPRPWSRNAAPSSTKRRDSKLWSETFDVCVNQMLTSKE
IKRQEAIFELSQGEEDLIEDLKLAKKAYHDPMLKLSIMTEQELNQIFGTLDSLIPLHEEL
LSQLRDVRKPDGSTEHVGPILVGWLPCLSSYDSYCSNQVAAKALLDHKKQDHRVQDFLQR
CLESPFSRKLDLWNFLDIPRSRLVKYPLLLREILRHTPNDNPDQQHLEEAINIIQGIVAE
INTKTGESECRYYKERLLYLEEGQKDSLIDSSRVLCCHGELKNNRGVKLHVFLFQEVLVI
TRAVTHNEQLCYQLYRQPIPVKDLLLEDLQDGEVRLGGSLRGAFSNNERIKNFFRVSFKN
GSQSQTHSLQANDTFNKQQWLNCIRQAKETVLCAAGQAGVLDSEGSFLNPTTGSRELQGE
TKLEQMDQSDSESDCSMDTSEVSLDCERMEQTDSSCGNSRHGESNV

>sp|Q91X46|ARHG3_MOUSE Rho guanine nucleotide exchange factor 3 OS=Mus musculus OX=10090 GN=Arhgef3 PE=1 SV=1
MVAKDYPFYLTVKRANCSLEAPLGSGVAKDEEPSNKRVKPLSRVTSLANLIPPVKTTPLK
RFSQTLQRSISFRSESRPDILAPRAWSRNATSSSTKRRDSKLWSETFDVCVNQVLTAKEI
KRQEAIFELSQGEEDLIEDLKLAKKAYHDPMLKLSIMTEQELNQIFGTLDSLIPLHEELL
SQLRDVRKPDGSTEHVGPILVGWLPCLSSYDSYCSNQVAAKALLDHKKQDHRVQDFLQRC
LESPFSRKLDLWNFLDIPRSRLVKYPLLLREILRHTPNDNPDQQHLEEAINIIQGIVAEI
NTKTGESECRYYKERLLYLEEGQKDSLIDSSRVLCCHGELKNNRGVKLHVFLFQEVLVIT
RAVTHNEQLCYQLYRQPIPVKDLTLEDLQDGEVRLGGSLRGAFSNNERVKNFFRVSFKNG
SQSQTHSLQANDTFNKQQWLNCIRQAKETVLSAAGQAGLLDSEGLVQGPGTENREPQGET
KLEQMDQSDSESDCSMDTSEVSLECERMEQTDASCANSRPEESV

>tr|Q1MT42|Q1MT42_DANRE Rho guanine nucleotide exchange factor 3 OS=Danio rerio OX=7955 GN=arhgef3 PE=4 SV=1 (Zebrafish)
MVAKDYPIYLLIKLANCSVDESSSCGRGVGRELEEPSTKRVKPLSRVTSLASLIPPVKAA
PLKRIGQTLQRSISFRSDCRPEIMAPRPWSRQVVPTITKRRDSKLWSETFDVGLGHMLSS
KEIKRQEAIFELSQGEQDLIEDLKLAKKAYHDPMLKLCIMTEHELNQIFGTLDSLIPLHE
DLLSRLRDVRNPDGSTDHMGHILVHWLPCLETYNSYCSNQVAAKALLDHKKQDHRVHDFL
QRCLESPFSRKLDLWNFLDIPRSRLVKYPLLLREILKHTPNEHPDRQHLDEAINMIQSIV
AGINTQTGESECRYYKERLLYLESGQRDLLIESSRILICHGELKNNRGVKLHVFLFQDVL
VITRAISHNEQLWYQLYRQPIPVRDLEWEDLQDGEMRVGGSIRGAFSNNERTKNFFRVMF
RCGGQTQMHCFQASDTFNKQQWLNCIRQAKEAVGSAESEPPRSPQMDRERQHNDCSDSNM
MDTMEDSSSEYPQMELADIPVSPVRE

>sp|Q5ZLX4|ARHG3_CHICK Rho guanine nucleotide exchange factor 3 OS=Gallus gallus OX=9031 GN=Arhgef3 PE=2 SV=1
MVAKDYPFYLTVKRANCALDVEAASSPAKETEEPSNKRVKPLSRVTSLANLIPPVRATPL
KRFSQTLQRSISFRSDSRPDLFSPRPWSRNTPAANTKRRDSKLWSETFDVCVNHMLTSKE
IKRQEAIFELSKGEEDLIEDLKLAKKAYHDPMLKLSIMTEQELNQIFGTLDSLIPLHEDL
LRRLQEVRKSDGSTEHVGHILVGWLPCLNSYDSYCSNQVAAKALLDHKKQDHRVQDFLQR
CLESPFSRKLDLWNFLDIPRSRLVKYPLLLREILRHTPNDHPDQQHLEEAINIIQGIVAE
INIKTGESECQYYKERLIYLEGGQRDSLIDNSRVLCCHGELKNNRGVKLHVFLFQEVLVI
TRAITHNEQLCYQLYRQPIPVMDLVLEDLQDGEVRLGGSIRGAFSNNERIKNFFRVSFKN
GSQSQSHSLQANDSFNKQQWLNCIRQAKEKVTCAGKAGVLSSEACFILSPNGSRVSQGET
MIEQMDQSDSESDCSMDTSEISIDCERMEQTNPCENEKQIETNV

>tr|Q32NJ3|Q32NJ3_XENLA Rho guanine nucleotide exchange factor 3 OS=Xenopus laevis OX=8355 GN=arhgef3.2.L PE=2 SV=1
MVAKDYPFYLTVKRVNLTLEVKTTRNPTKEIEEPSTKRVKPLSRVTSLANLIPPAKPTPL
KRFSQSLQRSISFRSDSRPDLFTPRPWSRNAPPPITKRRDSKLWSETFDVCVNQVLTSQE
IKRQEVIFELSQGEEDLIEDLKLAKKAYHDPMLKLSIMTEQELTQIFGTLDSLIPLHEDL
LRRLREVRKPDGSTENVGHILVGWLPCLNSYDSYCSNQVAAKALLDHKKQDHRVQDFLQR

CLESPFSRKLDLWNFLDIPRSRLVKYHLLLKELLRHTPNGHQDQQHLEEAINIVQGIVAE
INIKTGESECHYYKERLIYLDECQKDPLIDNSRALCCHGELKNNRGAKFHVFLFQDVLMI
TRTITQNEALYYQVYRQPIPVKDLILDDLQDGEVRLAGSLRGAFSNNERIKNFFRVGFKN
GSHSQAHFLQANDTFNKQQWLNCIRQAKESVVCSGGDDASSSNRRLSISANGTCGSQPPA
RFEKMDQSDSVSDCSMDISETSMDCEQMEQTNSHNKEEVESNV

>tr|F7CBC5|F7CBC5_XENTR Rho guanine nucleotide exchange factor (GEF) 3, gene 1 OS=Xenopus tropicalis OX=8364 GN=asb13 PE=4 SV=4
MATGGSIVRGVTAPRGCAMDESEGPEEKEETESWYGGQGQLGEELTPFWGRLVWGTGLKR
RKQVEPPPGQVAEEKEPSSKRVKPVSKTLSASVAPPPAKVTPLKKLSLSIQKSIGCKSES
RLFSFPPRTRSREPGGGAKRRSSKLWRETFDSAGEELSAREIKRQEVIFELMQGEHTLIE
DLNLVRKNYYEPMLKLEILSREELEQIFGILDSLAPLHQDLLERLLKLRREDGTLEVIGP
TMLDWLPCLHAYKTYCCNQVAAKSLLDHKRRCAAVQNFLRLCQESAFSRKLDVWSFLDLP
RSRLVKYPLLLKELLKHTGPEHPDYTPLGEAVCMIQTIVSLINSKTGEAECEYYRRRLTY
LYESQEEGAVTSSRLLHCHGELKNNKGQRLQVFLFEAALVLTRPITQNHQLQFQVYRQPI
PLQELVLEDLPDGEVRVGGSIRGAFTPATERAKNFFRVSFQDRSRGQSHTLQANDSFNKQ
QWVSCIRQAKAANQGGPPEPPCTLVDISDLSLSSIEDCEKMEDG

>tr|A0A670I4W0|A0A670I4W0_PODMU Rho guanine nucleotide exchange factor 3 OS=Podarcis muralis OX=64176 GN=ARHGEF3 PE=4 SV=1 (common wall lizard)
MGWCCFFVHHQKKRKQSAQSEDVISVCSFDSNEPSNKRVRPLSRVTSLASLIPPVRATPL
KRFGQTLQRSISFRSDSRPDLLASRPWTRNVPPASTKRRDSKLWSETFDVCVNQMLTSKE
IKRQEAIFELSQGEEDLIEDLKLAKKAYHDPMLKLSIMSEQELNQIFGTLDSLIPLHEDL
LRRLKEIRKPDGSTDCVGHILVGWLPCLNSYDSYCSNQVAAKTLLDQKKQDHRVQDFLQR
CLESPFSRKLDLWNFLDIPRSRLVKYPLLLREILRHTPNDHPDQQHIEEAINIVQGIVAE
INKKTGESECQYYKERLIYLDEGQKDSLIDDSRVVCCHGELKNNRGAKLHVFLFQEVLVI
TRAVTQNEQLCYQLYRQPIPVKDLVLEDLQDGEVRLGGSIRGAFSNNERIKNFFRVSFRK
GSQSQSHSLQANDTFNKQQWLNCIRQAKESVSCASSEIRTLDSEDPLLTSSSTSSSRNRI
PRQESKLEQMDQSDSGSDCSMDISEISIDCDHMEQTYPWQTDKEIESNV

>tr|A0A5F4CCC6|A0A5F4CCC6_CANLF Rho guanine nucleotide exchange factor 3 OS=Canis lupus familiaris OX=9615 GN=ARHGEF3 PE=4 SV=1 (Dog)
MVAKDYPFYLTVKRANCSLDVPPSSSPAKDLEEPSNKRVKPLSRVTSLANLIPPVKATPL
KRFSQTLQRSISFRSESRPDILAPRPWSRNAAPSSTKRRDSKLWSETFDVCVNQMLPSKE
IKRQEAIFELSQGEEDLIEDLKLAKKAYHDPMLKLSIMTEQELNQIFGTLDSLIPLHEEL
LSQLRDARKPDGSTEHVGPILVGWLPCLSSYDSYCSNQVAAKALLDHKKQDHRVQDFLQR
CLESPFSRKLDLWNFLDIPRSRLVKYPLLLREILRHTPNDNPDQQHLEEAINIIQGIVAE
INTKTGESECRYYKERLLYLEEGQKDSLIDNSRVLCCHGELKNNRGVKLHVFLFQEVLVI
TRAVTHNEQLCYQLYRQPIPVKDLLLEDLQDGEVRLGGSLRGAFSNNERIKNFFRVSFKN
GSQSQTHSLQANDTFNKQQWLNCIRQAKETVLCAAGQAGDLDSEGPFLNPATGSREPQGE
TKLEQMDQSDSESDCSMDTSEVSLDYERMEQTDSSCGNSRHVESNV
