# Supplementary material for: Bmp Signal Gradient Modulates Convergent Cell Movement via Xarhgef3.2 during Gastrulation of Xenopus Embryos
Source: Cells. 2021 Dec 24;11(1):44. doi: 10.3390/cells11010044 (PMC8750265; doi:10.3390/cells11010044)
Supplement: Supplementary file 1 [file cells-11-00044-s001.zip › cells-1447007-Supplementary figures and tables.pdf]

Bmp Signal Gradient Modulates Convergent Cell Movement via *Xarhgef3.2* during Gastrulation of *Xenopus* embryos

Jaeho Yoon [1,2], Vijay Kumar [1], Ravi Shankar Goutam [1], Sung-Chan Kim [1], Soochul Park [3], Unjoo Lee [4] and Jaebong Kim [1,*]

[1] Department of Biochemistry, Institute of Cell Differentiation and Aging, College of Medicine,
Hallym University, Chuncheon 24252, Korea; jaeho.yoon@nih.gov (J.Y.); vijay10187@gmail.com (V.K.); ravi2005gautam@gmail.com (R.S.G.); biokim@hallym.ac.kr (S.-C.K.)

[2] National Cancer Institute, Frederick, MD 21702, USA

[3] Department of Biological Sciences, Sookmyung Women's University, Seoul 04310, Korea; scpark@sookmyung.ac.kr

[4] Department of Electrical Engineering, Hallym University, Chuncheon 24252, Korea; ejlee@hallym.ac.kr

* Correspondence: jbkim@hallym.ac.kr

A

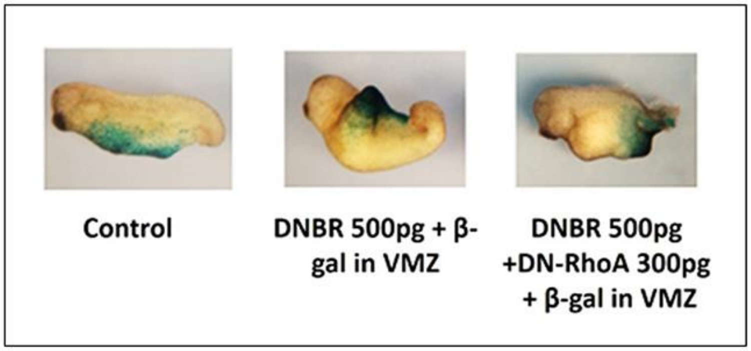
Control
DNBR 500pg + β-gal in VMZ
DNBR 500pg +DN-RhoA 300pg + β-gal in VMZ

B

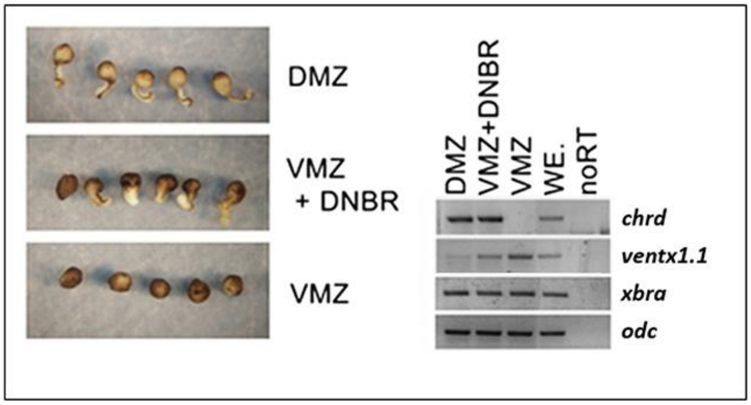
DMZ
VMZ + DNBR
VMZ
DMZ
VMZ+DNBR
VMZ
W.E.
noRT
chrd
ventx1.1
xbra
odc

C

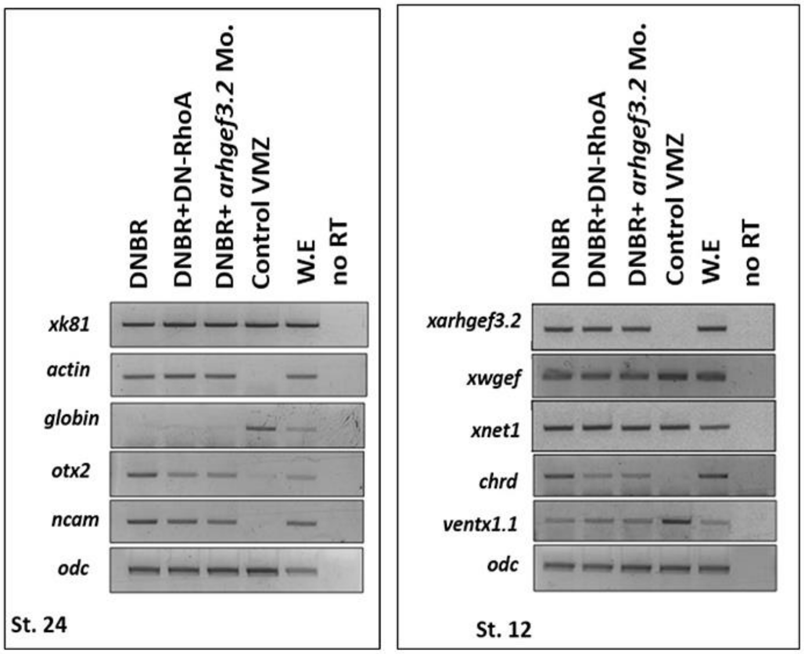
DNBR
DNBR+DN-RhoA
DNBR+ arhgef3.2 Mo.
Control VMZ
W.E
no RT
xk81
actin
globin
otx2
ncam
odc
St. 24
DNBR
DNBR+DN-RhoA
DNBR+ arhgef3.2 Mo.
Control VMZ
W.E
no RT
xarhgef3.2
xwgef
xnet1
chrd
ventx1.1
odc
St. 12

Figure S1. Dominant negative form of RhoA (DN-RhoA) suppressed the secondary axis formation induced by DNBR. (**A**)**,** Embryos were co-injected with DNBR (500 pg) with or without DN-RhoA (300 ng) into the VMZ of the 4 cell stage embryos. The morphological changes were analyzed at the 28-30 stages. β-gal staining was used for tracing the injected cell lineage. (**B**)**,** Convergence and extension (CE) in Keller explants and the gene expressions were analyzed in the dissected DMZ and VMZ samples (indicated) (B; right panel). (**C**)**,** Gene expression analysis in the condition of DNBR with DN-RhoA. DNBR mRNAs (500 pg) were co-injected with DN-RhoA (300 ng) or ARHGEF3.2 morpholino oligos (20 ng) into VMZ of the 4 cell stage embryos. The VMZ explants were dissected at stage 10 and then incubated in 1X L-15 growth medium until stage 12 and 24 to perform RT-PCR analysis.

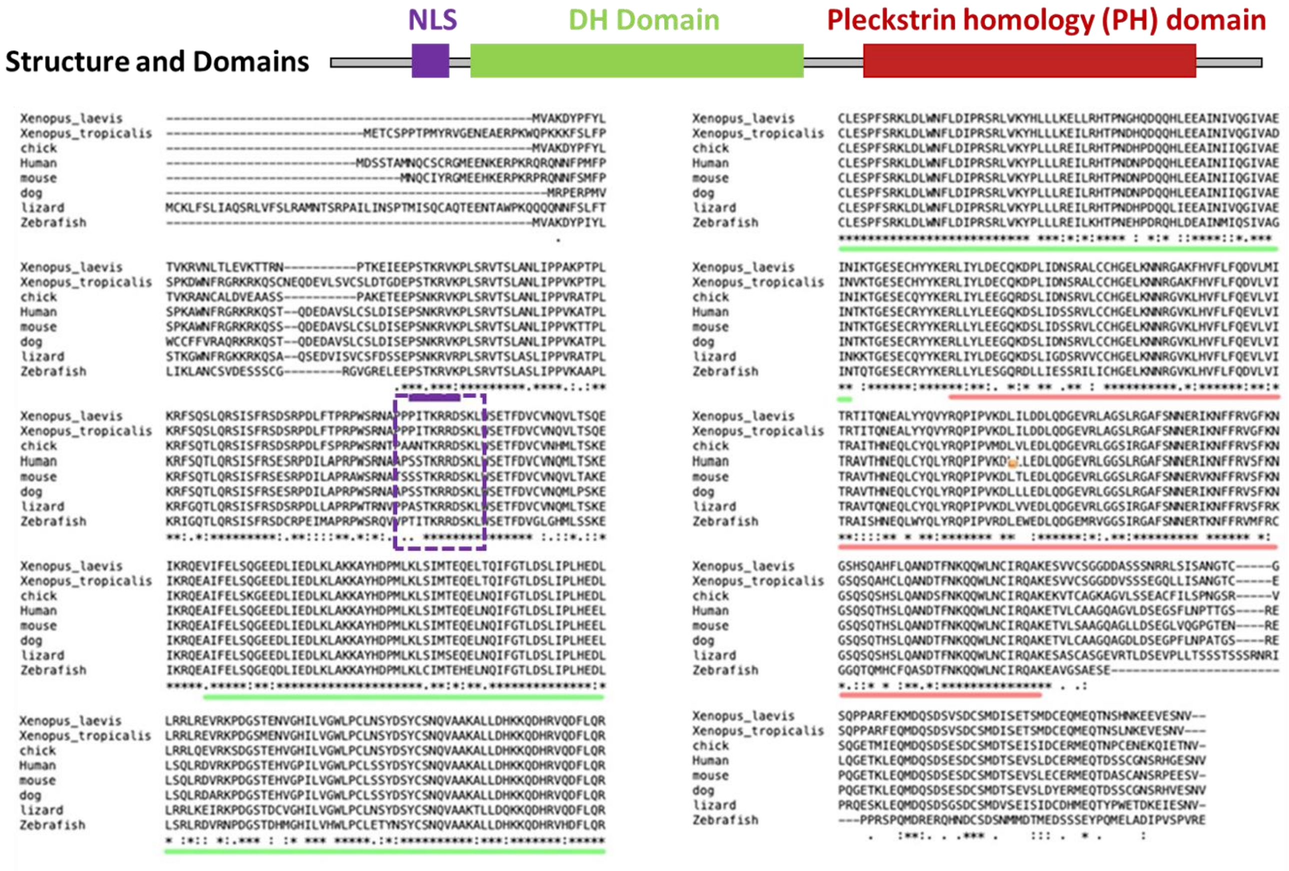

Figure S2. Alignment of Xenopus arhgef3.2 sequences with human, chick, lizard, zebrafish and mouse ARHGEF3 sequences. The nuclear localization signal, NLS is indicated by the purple line (dotted box). The Dbl-homology (DH) and pleckstrin-homology (PH) domains are also indicated by green and red under lines, respectively.

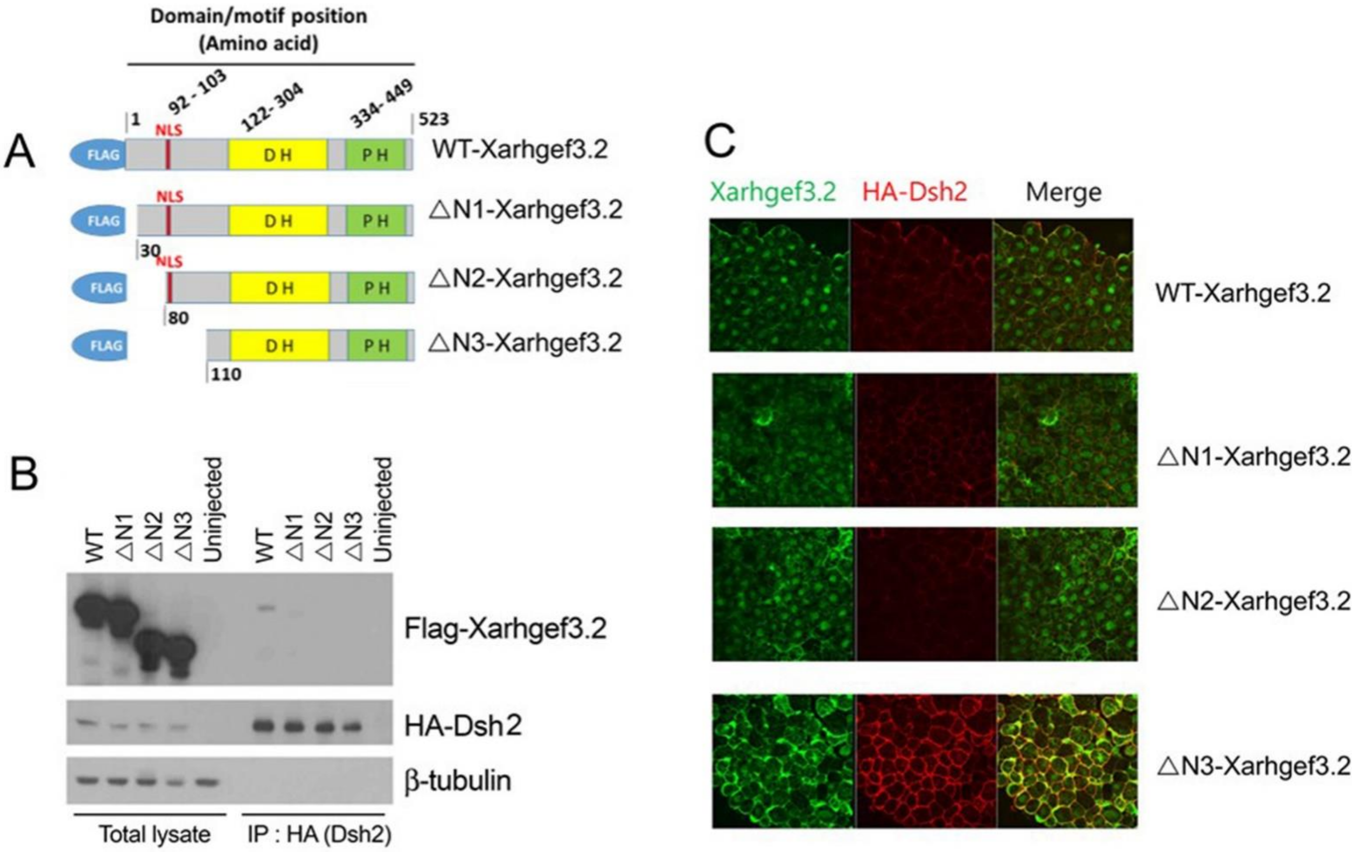

Figure S3. Truncated nuclear localization signal (NLS) block Arhgef3.2 nuclear localization. (**A**) The structure of Xarhgef3.2 and different NLS truncated constructs (ΔN1, ΔN2, ΔN3). (**B**) Immunoprecipitation of NLS truncated constructs and interaction with Dsh. (**C**) RNAs of NLS truncated *Flag-Xarhgef3.2* (100 pg/embryo) and *HA-Dsh* (100 pg/embryo) were co-injected into dorsal marginal zone at the 4 cell stage. DMZ explants were dissected at stage 10 and fixed using MEMFA and stained with anti-Flag and anti-HA antibodies. Localization of Flag-Xarhgef3.2 was analyzed with DMZ explants using confocal microscopy. The truncated constructs lacking first 29, 79, and 109 amino acids in ΔN1, ΔN2, ΔN3-Xarhgef3.2 respectively)

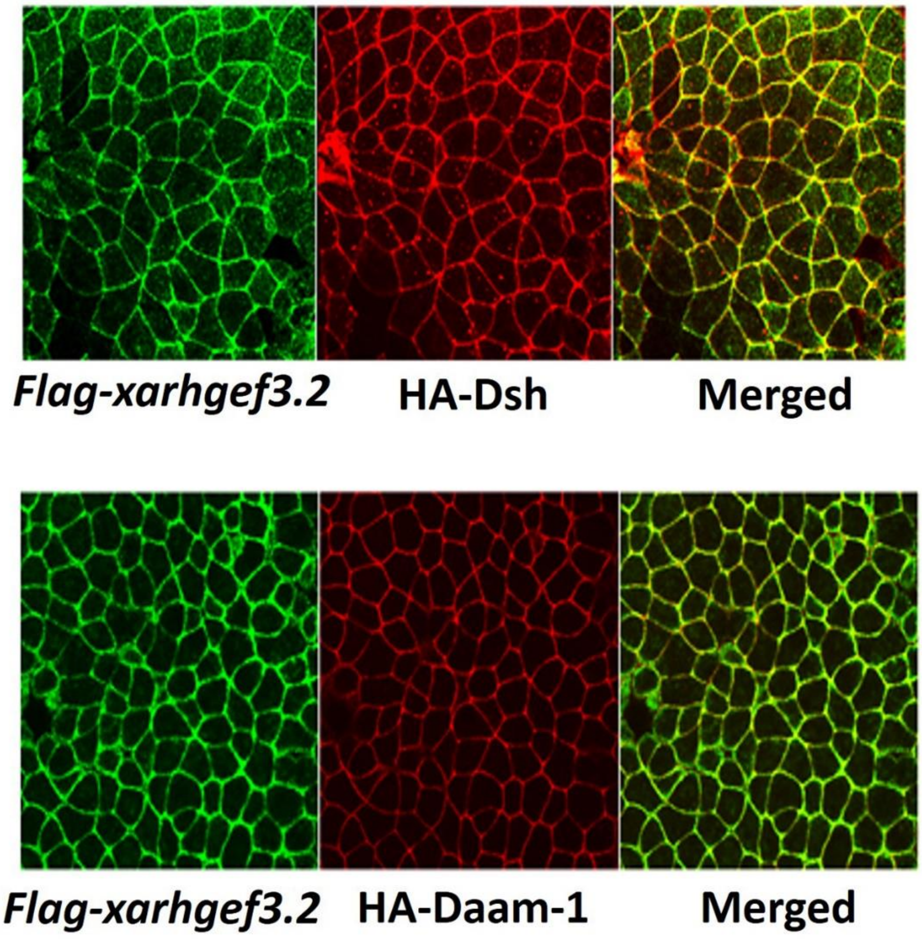

Figure S4. Arhgef3 is co-localized with Dsh and Daam-1. RNAs of Flag-arhgef3 (100 pg) and HA-Dsh or HA-Daam-1 (200 pg) were co-injected into DMZ at the 4 cell stage. DMZ explants were dissected at stage 10, fixed using MEMFA and then stained with anti-Flag and HA antibodies. Localization of arhgef3, Dsh and Daam-1 were studied with confocal microscopy on Zeiss LSM 510. Control DMZ explants that were dissected from un-injected embryos showed no specific staining with anti-Flag and HA antibodies (data not shown).

Table S1:

Normalized and analyzed microarray data of upregulated genes from activin treatment.

Txt file with columns consisting of list number, affymetrix probe ID, signal of control, compared signal of control vs. selected genes, log ratio of control vs. selected genes, gene symbol and gene name. These were assigned by the Affymetrix *Xenopus* Genome Gene Chip analysis.

Table S2:

Normalized and analyzed microarray data of upregulated genes from Dnbr treatment.

Txt file with columns consisting of list number, affymetrix probe ID, signal of control, compared signal of control vs. selected genes, log ratio of control vs. selected genes, gene symbol and gene name. These were assigned by the Affymetrix *Xenopus* Genome Gene Chip analysis.

Table S3:

Normalized and analyzed microarray data of upregulated genes from Fgf treatment.

Txt file with columns consisting of list number, affymetrix probe ID, signal of control, compared signal of control vs. selected genes, log ratio of control vs. selected genes, gene symbol and gene name. These were assigned by the Affymetrix *Xenopus* Genome Gene Chip analysis.
